# Characterization of *sucrose nonfermenting-1-related protein kinase 2* (*SnRK2*) gene family in *Haynaldia villosa* demonstrated *SnRK2.9-V* enhances drought and salt stress tolerance of common wheat

**DOI:** 10.1186/s12864-024-10114-7

**Published:** 2024-02-26

**Authors:** Jia Liu, Luyang Wei, Yirong Wu, Zongkuan Wang, Haiyan Wang, Jin Xiao, Xiue Wang, Li Sun

**Affiliations:** 1 National Key Laboratory of Crop Genetics & Germplasm Enhancement and Utilization, Cytogenetics Institute, Zhongshan Biological Breeding Laboratory, Nanjing Agricultural University/JCIC-MCP, Nanjing, 210095 China; 2 Jinhua Academy, Zhejiang Chinese Medical University, Jinhua, 321000 China

**Keywords:** *Haynaldia villosa* L., *Triticum aestivum* L., *SnRK2*, Gene family, Expression profiling, Abiotic stress

## Abstract

**Background:**

The sucrose nonfermenting-1-related protein kinase 2 (SnRK2) plays a crucial role in responses to diverse biotic/abiotic stresses. Currently, there are reports on these genes in *Haynaldia villosa*, a diploid wild relative of wheat*.*

**Results:**

To understand the evolution of *SnRK2-V* family genes and their roles in various stress conditions, we performed genome-wide identification of the *SnRK2-V* gene family in *H. villosa*. Ten *SnRK2-V* genes were identified and characterized for their structures, functions and spatial expressions. Analysis of gene exon/intron structure further revealed the presence of evolutionary paths and replication events of *SnRK2-V* gene family in the *H. villosa*. In addition, the features of gene structure, the chromosomal location, subcellular localization of the gene family were investigated and the phylogenetic relationship were determined using computational approaches. Analysis of cis-regulatory elements of *SnRK2-V* gene members revealed their close correlation with different phytohormone signals. The expression profiling revealed that ten *SnRK2-V* genes expressed at least one tissue (leave, stem, root, or grain), or in response to at least one of the biotic (stripe rust or powdery mildew) or abiotic (drought or salt) stresses. Moreover, *SnRK2.9-V* was up-regulated in *H. villosa* under the drought and salt stress and overexpressing of *SnRK2.9-V* in wheat enhanced drought and salt tolerances via enhancing the genes expression of antioxidant enzymes, revealing a potential value of *SnRK2.9-V* in wheat improvement for salt tolerance.

**Conclusion:**

Our present study provides a basic genome-wide overview of *SnRK2-V* genes in *H. villosa* and demonstrates the potential use of *SnRK2.9-V* in enhancing the drought and salt tolerances in common wheat.

**Supplementary Information:**

The online version contains supplementary material available at 10.1186/s12864-024-10114-7.

## Background

Wheat (*Triticum aestivum* L.) is an important food crop providing essential nutrients to human beings [1], and its production is crucial to ensuring global food supply security [2, 3]. However, the abiotic stresses, such as drought and salt, threatened wheat production by reducing its grain yield and quality [3–5]. Therefore, identification of genes regulating abiotic stress tolerances and the elucidation of their regulatory mechanisms are of great significance for the improvement of wheat tolerances to various abiotic stresses via a molecular approach.

The phytohormone abscisic acid (ABA) signaling pathway plays an important role in the tolerant or adaptive responses to droughts, salinization, and other environmental stresses [6], and the sucrose nonfermenting-1-related protein kinase 2 (SnRK2) family members act as crucial regulators in enhancing abiotic stresses tolerance or adaptability [6, 7]. Based on the homology analysis of amino acid sequence, the SnRK2s can be classified into three sub-classes, namely Group I, Group II and Group III. Under ABA treatment, the expression levels of *SnRK2s* are obviously up-regulated in Group III and slightly induced in Group II, but not in Group I [8]. *SnRK2* genes in Group III have been identified and characterized, with three in *Arabidopsis thaliana* (*AtSnRK2.2*, *AtSnRK2.3, AtSnRK2.6*) [8], three in *Oryza sativa* (*OsSAPK8*, *OsSAPK9*, *OsSAPK10*) [9], three in *Zea mays* (*ZmSnRK2.8, ZmSnRK2.9, ZmSnRK2.10*) [10, 11], three in *Brachypodium distachyon* (*BdSnRK2.8*, *BdSnRK2.9*, *BdSnRK2.10*) [12], four in *Malus prunifolia* (*MpSnRK2.1*, *MpSnRK2.8*, *MpSnRK2.9*, *MpSnRK2.10*) [13] and three in *Camellia sinensis* (*CsSnRK2.5*, *CsSnRK2.6*, *CsSnRK2.7*) [14]. Similarly, six Group III *SnRK2* genes from Triticeae species have been reported after induction of ABA treatment, including *HvSnRK2.8, HvSnRK2.9* and *HvSnRK2.10* in *Hordeum vulgare* [15], *TaSnRK2.8*, *TaSnRK2.9* and *TaSnRK2.10* in *T. aestivum* [16].

Accumulating evidence has documented that Group III SnRK2s are well characterized as key positive regulators in salt and drought tolerances. For example, the expression levels of *AtSnRK2.2, AtSnRK2.3* and *AtSnRK2.6* increased under salt and drought stresses [17]. The *srk2d/e/i* triple mutant (for *AtSnRK2.2*, *AtSnRK2.6* and *AtSnRK2.3*) dramatically displayed the decreased drought tolerance [18]. *OsSAPK8*, *OsSAPK9* and *OsSAPK10* were up-regulated under salt stress [9, 19–21], in which *OsSAPK8* and *OsSAPK9* were also activated by drought stress [19, 20]. Of note, knockout of *OsSAPK8* showed lower tolerances to high salinity and drought stresses [19], and overexpression of *OsSAPK9* improved the tolerance of rice to drought stress [20]. Similarly, *TaSnRK2.8* and *TaSnRK2.9* were up-regulated by salt and drought stresses [22–24]. Notably, heterologous overexpression of *TaSnRK2.8* in Arabidopsis or *TaSnRK2.9* in tobacco enhanced their tolerances to high salinity and drought stresses [22–24]. Interestingly, overexpression of *TaSnRK2.8* in *Arabidopsis* grew longer primary roots [23], and transgenic tobacco overexpressing *TaSnRK2.9* improved root length [24]. In addition, the transcript of *ZmSnRK2.8* increased under high salinity and drought stresses, and heterologous overexpression of this gene in the Arabidopsis also significantly strengthened its tolerance to salt stress [25]. Moreover, *MpSnRK2.10* was dramatically induced by drought, and overexpression of this gene enhanced drought tolerance of apples and Arabidopsis [26].

Under drought-or high salt-stress conditions, reactive oxygen species (ROS) accumulated in plants and brought about oxidative damage and programmed cell death [27]. Compelling molecular evidence revealed that the increased transcriptions of *SnRK2s* can increment the transcripts of antioxidant enzymes genes, such as *superoxide dismutase* (*SODs*), *ascorbate peroxidase* (*APXs*), *catalase* (*CATs*), *peroxidase* (*PODs*), *glutathione* (*GSHs*) and/or corresponding protein levels, which are involved in scavenging ROS. Consequently, they relieved oxidative stress to decrease malondialdehyde (MDA) and kept normal growth and development of plant under different stresses [28, 29]. Under high salinity tolerance, *AtSnRK2.4* and *AtSnRK2.10* regulated the expression of *CATs* and *APXs* that responsible for ROS homeostasis [28, 29]. Conversely, overexpression of *OsSAPK1* and *OsSAPK2* promoted the production of ROS scavenger (ascorbic acid) and increased the protein levels of SOD and CAT, leading to the improved ROS detoxification [30, 31]*.* Of note, *Ossapk2* mutants were more sensitive to drought stress, with the remarkable increased transcript levels of *OsCAT*, *OsCu/Zn-SOD1*, *OsCu/Zn-SOD2* and *OsAPX2* [30, 31]. Recently, it has been demonstrated that heterologous overexpression of *TaSnRK2.9* in tobacco enhanced drought and high salt tolerances via reducing H_2_O_2_ content by SOD, CAT, POD and GSH [24]. More recently, overexpressing *CsSnRK2.5* in Arabidopsis has been reported to enhance drought tolerance by decreasing accumulation of ROS and MDA [32].

*Haynaldia villosa* L. (2n = 2x = 14, VV), a diploid wild relative of wheat, is a valuable genetic resource harboring many elite traits, such as resistance to tolerance to various abiotic stresses [33–35]. Simultaneously, *SnRK2* gene family plays an important role in different abiotic stresses, however, there is still a sustained lack of research on understanding the functional import of the *SnRK2* genes in *H. villosa*. Herein, we aimed to elucidate the evolution and diversification of *SnRK2-V* genes and potential roles of *SnRK2.9-V* in drought and high salinity stresses. First, different members of *SnRK2-V* genes were identified in *H. villosa,* and phylogenetic tree and evolutionary relationship of *SnRK2-V* genes were analyzed. Secondly, chromosome distribution and gene structure were further studied to gain a better understanding of *SnRK2-V* genes, in which ten genes were cloned from *H. villosa* and their potential functions were elucidated by quantitative RT-PCR (qRT-PCR). Finally, *SnRK2.9-V* was transformed into common wheat to analyze its role upon salt and drought stresses. In a word, our results may provide new candidate genes for wheat to improve the tolerance of drought and soil salinization.

## Results

### Identification of *SnRK2-V* gene family in *H. villosa* and analysis of phylogenetic relationship

**Fig. 1:**
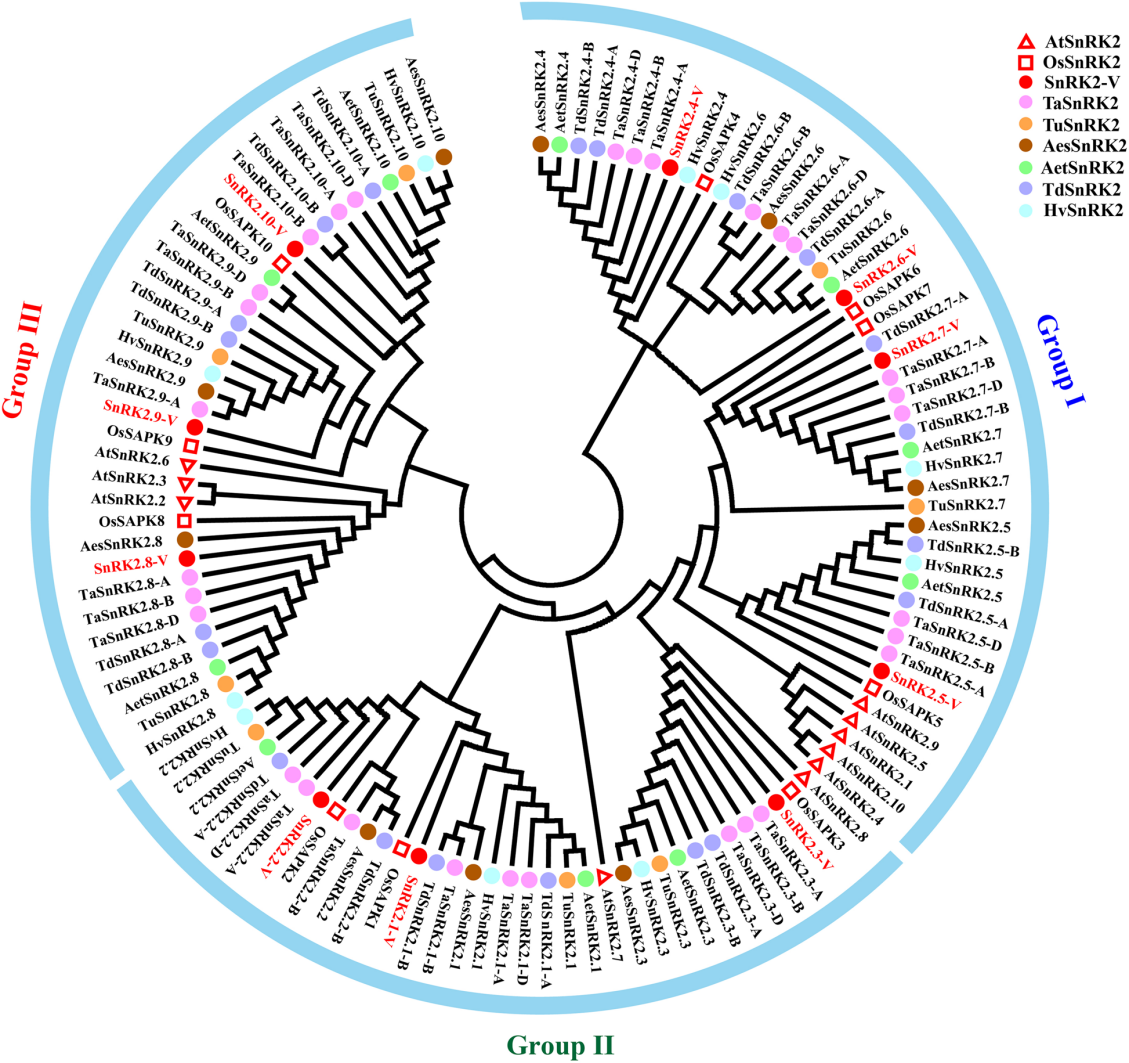
Phylogenetic tree of SnRK2 proteins from nine plant species. Tree was constructed by MEGA8.0 using neighbor-joining (NJ) method with 1000 bootstraps. SnRK2 protein sequences from *A. thaliana* (*At*), *Oryza sativa* (*Os*), *H. villosa (-V*)*, T. aestivum* (*Ta*), *T. urartu* (*Tu*), *Ae. speltoides* (*Aes*), *Ae. tauschii* (*Aet*), *dicoccoides* (*Td*), *H. vulgare* (*Hv*) were denoted by red triangle, red square, red circle, pink circle, orange circle, brown circle, green circle, blue circle and lightblue circle, respectively

Out of 98 *SnRK2* genes from seven Triticeae species, thirty *SnRK2* members were characterized in *T. aestivum*, eight in *T. urartu*, ten in *Aegilops speltoides*, ten in *Ae. tauschii*, twenty in *T. dicoccoides*, ten in *H. vulgare*, and ten in *H. villosa* genome sequence*.* These genes were named from *SnRK2.1-V* to *SnRK2.10-V* according to the phylogenetic relationship to wheat *SnRK2* genes (Fig. 1).

Multiple full-length protein sequence alignments of the identified *SnRK2s* with ten rice *OsSnRK2s* and ten Arabidopsis *AtSnRK2s* suggested that the ten *H. villosa SnRK2*-*V* could be classified into three subgroups, namely Group I (not induced by ABA), Group II (weakly induced by ABA), Group III (induced remarkably by ABA) (Fig. 1; Table S1). Group I contained SnRK2.4-V, SnRK2.5-V, SnRK2.6-V and SnRK2.7-V; Group II included SnRK2.1-V, SnRK2.2-V and SnRK2.3-V and Group III had SnRK2.8-V, SnRK2.9-V and SnRK2.10-V.

### Chromosomal distribution and duplication of *SnRK2-V* genes

**Fig. 2:**
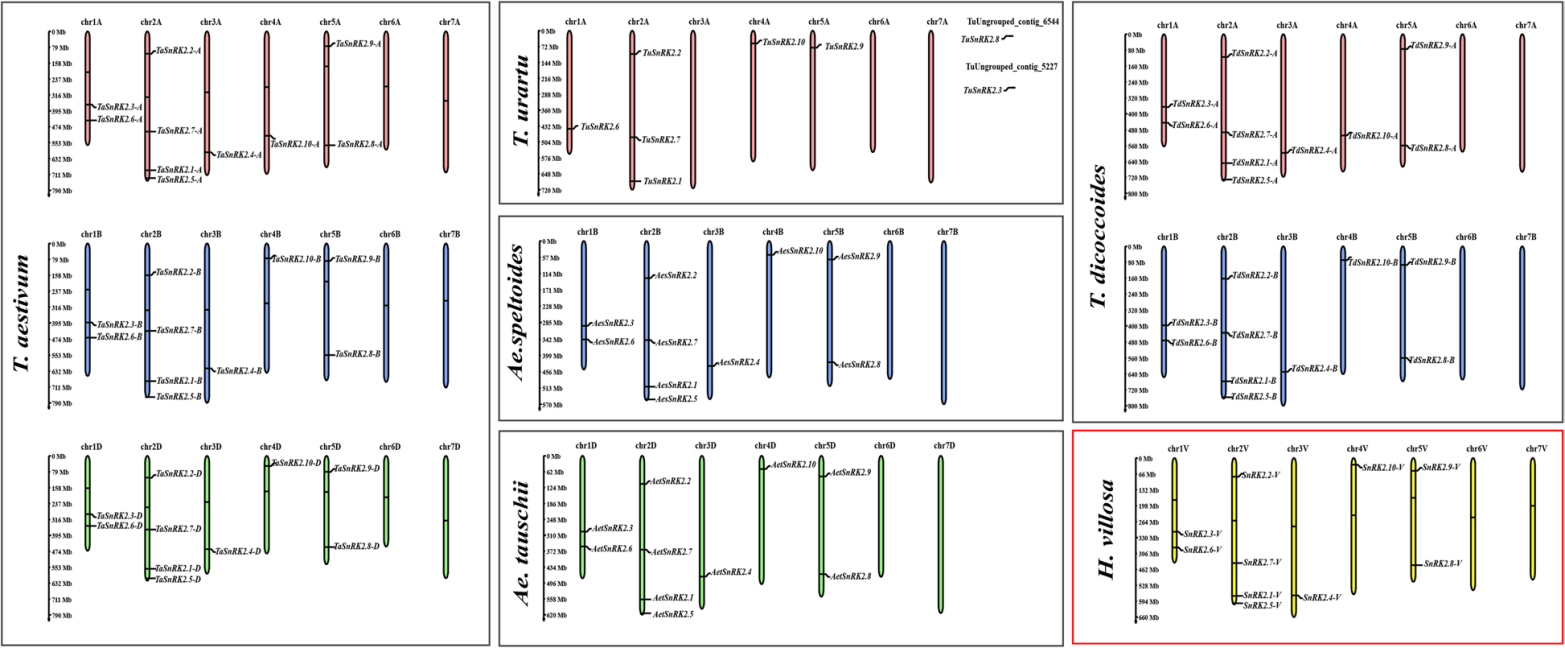
Chromosomal distribution of *SnRK2* genes in *T. aestivum*, *T. urartu*, *Ae. Speltoides*, *Ae. Tauschii*, *T. dicoccoides* and *H. villosa*. The *SnRK2* gene names were shown in the right of each chromosome. Species abbreviations: *Ta, T. aestivum; Tu, T. urartu; Aes, Ae. speltoides; Aet, Ae. tauschii; Td, T. dicoccoides; -V, H. villosa*

**Fig. 3:**
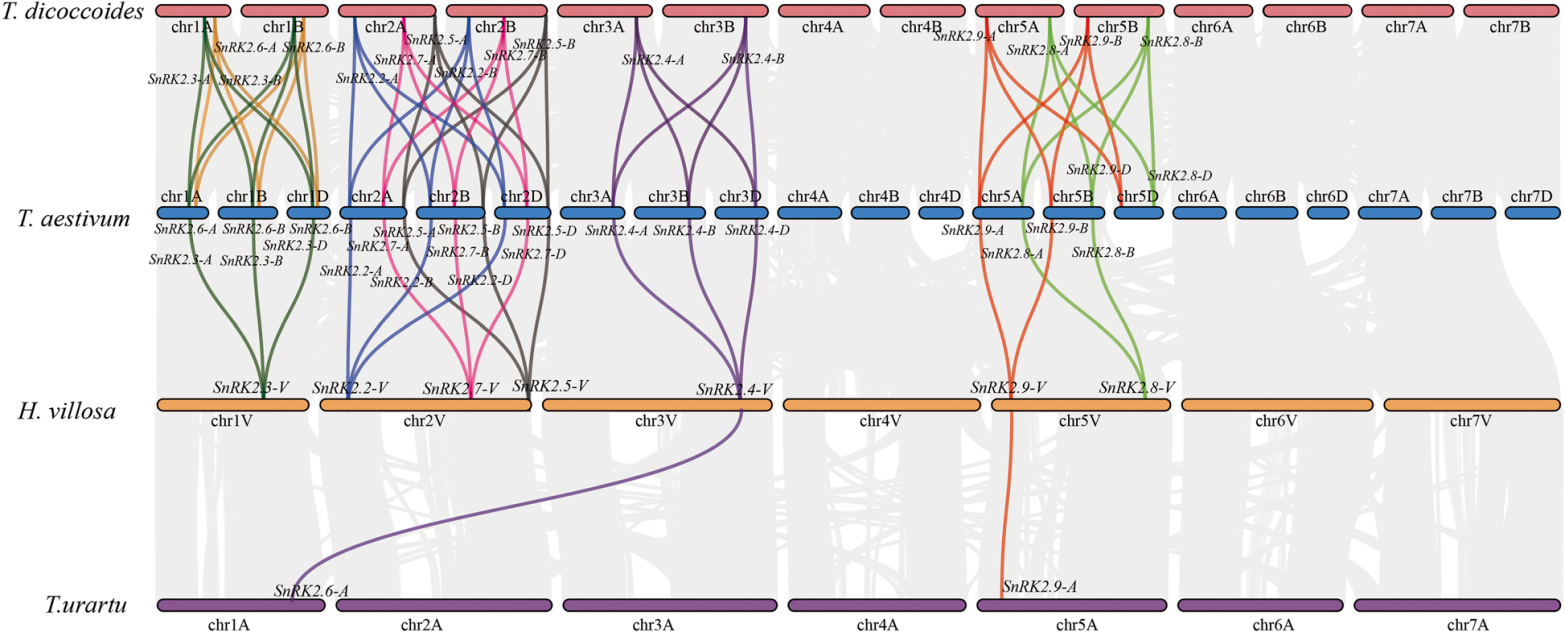
Syntenic relationships of *SnRK2-V* genes among *T. urartu*, *T. dicoccoides* and *T. aestivum*. Gray lines in the background indicated the collinear blocks within genome. Blue, dark green, purple, black, yellow, pink, green and red lines indicated the *SnRK2.2*, *SnRK2.3*, *SnRK2.4*, *SnRK2.5*, *SnRK2.6*, *SnRK2.7*, *SnRK2.8*, *SnRK2.9* collinear gene pairs, respectively

For chromosomal distribution, *SnRK2-V* genes were mapped to the corresponding chromosomes according to the completed genomic sequence of *H. villosa* in our previous study [36]. Ten *SnRK2-V* genes were identified and they were distributed unevenly on chromosomes. The maximum numbers on the chromosome 2 V were four genes namely *SnRK2.1-V, SnRK2.2-V, SnRK2.5-V* and *SnRK2.7-V*, which are similar to their homologous genes on the Group two chromosomes in five Triticeae species, except *SnRK2.5*. Chromosomes 3 V and 4 V only have one *SnRK2-V* gene each, *SnRK2.4-V* and *SnRK2.10-V* respectively, which are also in accordance with the homologous genes of five Triticeae species. In addition, *SnRK2.8-V* and *SnRK2.9-V* located on chromosome 5 V were consistent with their homologous genes on the Group five chromosomes except *T. urartu*. Similarly, *SnRK2.3-V* and *SnRK2.6-V* located on chromosome 1 V were consistent with that on the Group one chromosomes. Conversely, like all the Triticeae species, *SnRK2-V* genes were not present on chromosome 6 V and 7 V (Fig. 2). As shown in Fig. 3, *SnRK2.2-V, SnRK2.3-V, SnRK2.4-V, SnRK2.5-V* and *SnRK2.7-V* were separately collinear with *TaSnRK2.2-A, -B, -D, TaSnRK2.3-A, -B, -D, TaSnRK2.4-A, -B, -D, TaSnRK2.5-A, -B, -D* and *TaSnRK2.7-A, -B, -D*, respectively as well as the collineation with *T. dicoccoides*. However, the collinear gene pairs of *SnRK2.8-V* and *SnRK2.9-V* only existed in *TaSnRK2.8-A*, *-B* and *TaSnRK2.9-A*, *-B*, respectively. *SnRK2.1-V* had no collinear pairs with three subgenomes of *T. aestivum*, or same collinear pairs with *T. dicoccoides*. Notably, *SnRK2.9-V* and *SnRK2.4-V* had collinear gene pairs with *TuSnRK2.9-A*, and *TuSnRK2.6-A*, respectively, while the remaining *SnRK2-V* genes did not find the corresponding collinear relationship to that in *T. urartu* (Fig. 3).

### Gene structure and conserved motif analysis of *SnRK2-V* genes

**Fig. 4:**
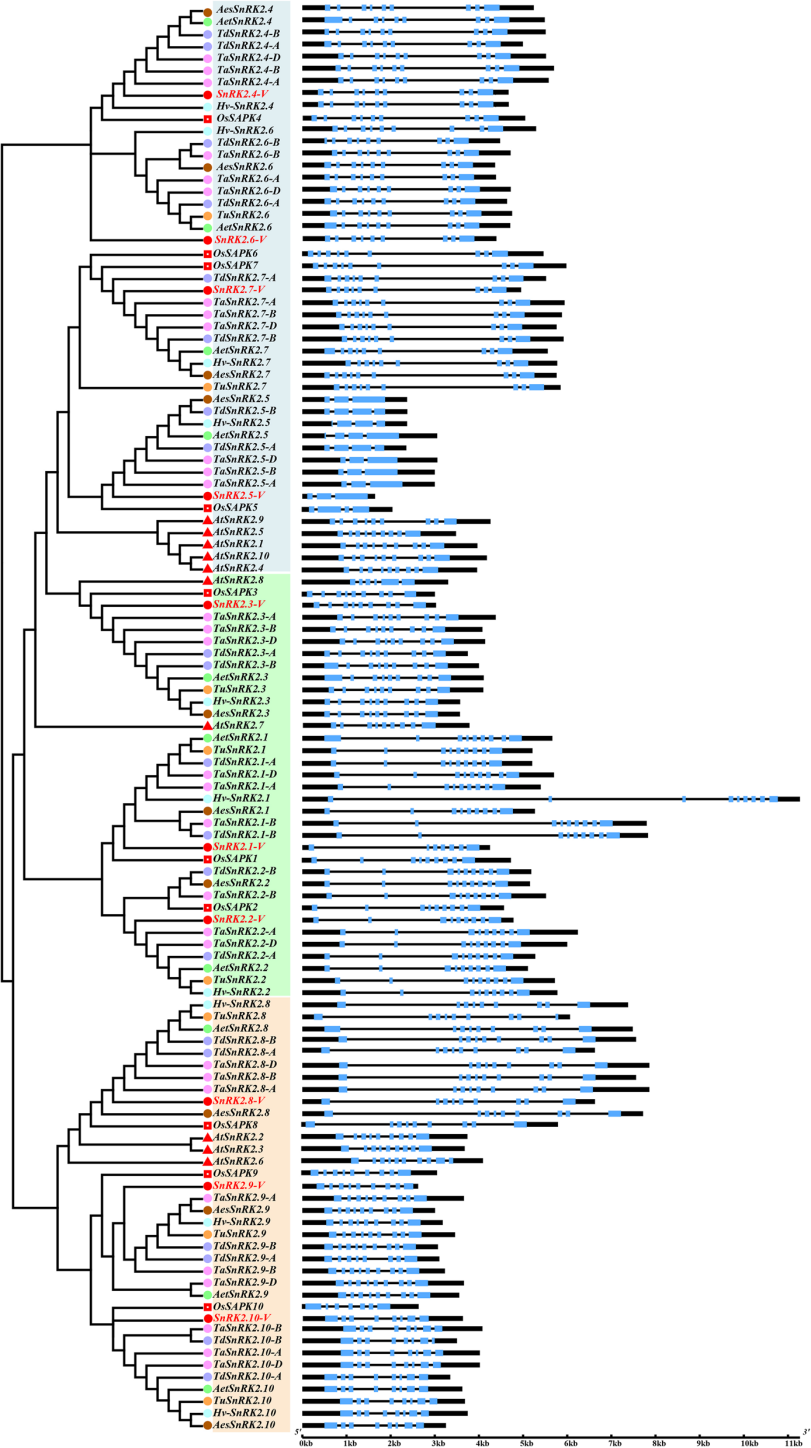
Exon–intron organization of *SnRK2* genes of nine plant species. Blue boxes represent exons, untranslated regions (UTRs) were indicated by black boxes and black lines represent introns. The exon and intron sizes were estimated using the scale at the bottom. The blue, green and red lines represented the Group I, Group II and Group III respectively. Species abbreviations: *At*: *A. thaliana*; *Os*: *O. sativa*; *-V*: *H. villosa*; *Ta*: *T. aestivum*; *Tu*: *T. urartu*; *Aes*: *Ae. speltoides*; *Aet*: *Ae. tauschii*; *Td*: *T. dicoccoides*; *Hv*: *H. vulgare*

Crystal structure analysis of *SnRK2-V* demonstrated that there were significant differences in the structures of *SnRK2-V* genes among three subgroups or within individual subgroup. Firstly, the number of introns varied significantly in Group I. Maximal number of intron was eight in *SnRK2.4-V*, *SnRK2.6-V* and *SnRK2.7-V*, which were the same as their orthologues in other Triticeae species and rice, e.g. *SAPK4*, *SAPK6* and *SAPK7* (Fig. 4). Conversely, at least two introns were detected in *SnRK2.5-V* gene, and a similar result was observed in *TaSnRK2.5-A*, *TaSnRK2.5-B* and *TaSnRK2.5-D* genes (Fig. 4). Simultaneously, except *SnRK2.1-V* containing six introns, eight introns were detected in *SnRK2.2-V* and *SnRK2.3-V* genes in Group II, and a similar result was observed in *SnRK2.8-V* and *SnRK2.9-V* genes in Group III (Fig. 4).

Secondly, the length of every intron varied dramatically and the gene length of each *SnRK2-V* gene was significantly different. The longest intron of the *SnRK2.8-V* was eight kb, whereas the shortest intron of the *SnRK2.5-V* gene was only three kb and the rest *SnRK2-V* genes varied from four kb to seven kb. Of note, the coding DNA sequence (CDS) length of *SnRK2-V* genes did not change remarkably, varying from 1,026 bp (*SnRK2.3-V*) to 1,179 bp (*SnRK2.5-V*) (Table S1).

**Fig. 5:**
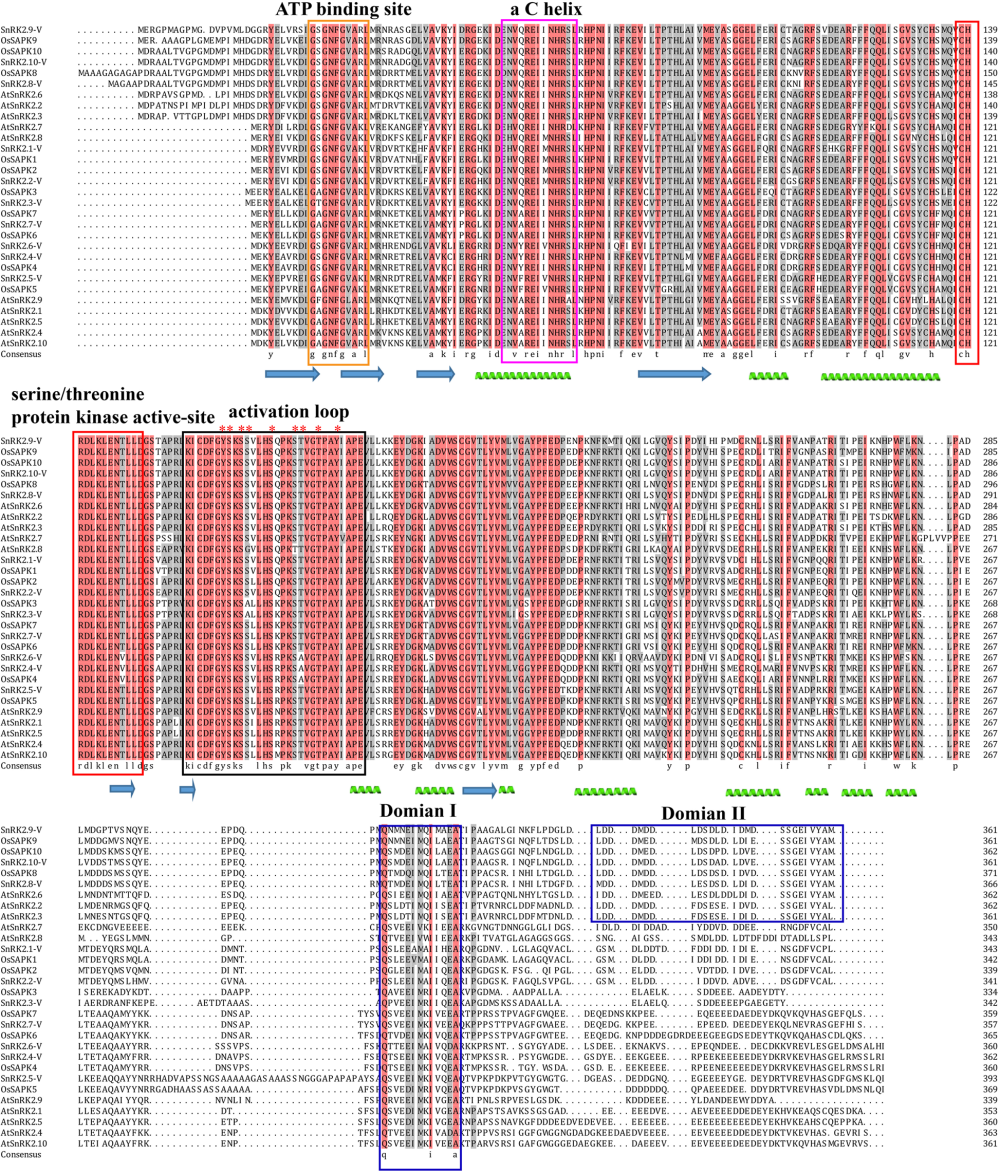
Protein sequence analysis of SnRK2 in *H. villosa*. ATP binding site, alpha C helix, serine/threonine protein kinase active-site, activation loop and SnRK2 Domain were shown in yellow box, magenta box, red box, black box and blue box respectively. Alpha helix was shown in green helix. Beta strand was shown in blue arrows. The predicted phosphorylation sites were indicated by red asterisks. Species abbreviations: *At*: *A. thaliana*; *Os*: *O. sativa*; *-V*: *H. villosa*

**Fig. 6:**
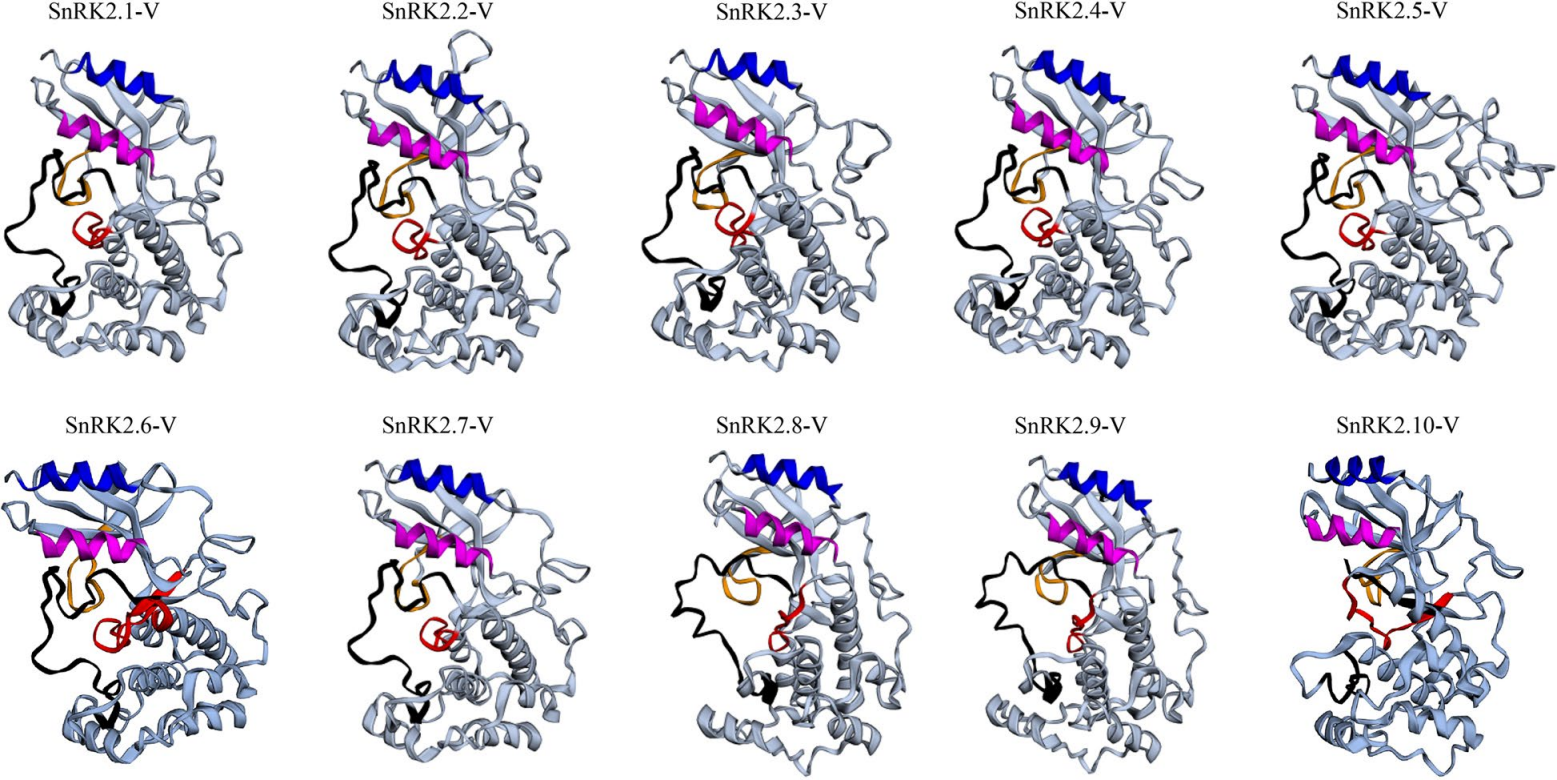
The predicted 3D structure of SnRK2-V proteins. ATP binding sites, Alpha C helixs, Serine/threonine protein kinase active-sites, Activation loops and SnRK2 boxs (domain I) were shown in yellow, magenta, red, black and blue, respectively

Finally, the amino acid lengths were from 342 aa (SnRK2.3-V) to 393 aa (SnRK2.5-V), with molecular weights ranging from 38.67 kDa (SnRK2.3-V) to 44.07 kDa (SnRK2.5-V). The isoelectric points of SnRK2-V family ranged from 4.80 (SnRK2.10-V) to 6.14 (SnRK2.5-V). All these identified SnRK2-V were predicted to be subcellularly localized in cytoplasm and nucleus (Table S1). Multiple sequence alignment displayed that the SnRK2-V proteins were highly conserved at the N-terminal containing an ATP binding site, alpha C helix, serine/threonine protein kinase active-site and activation loop, therefore they varied significantly at their C-terminal. All SnRK2-V proteins have domain I, which is associated with osmotic stress-mediated activation. The SnRK2.8-V, SnRK2.9-V and SnRK2.10-V proteins were different from other members due to their domain II, which is present only in the strongly ABA-responsive kinases. And each of the SnRK2-V proteins contained both alpha helix and beta strand (Fig. 5). Swiss-model indicated the homology modeling of ten SnRK2-V proteins three-dimensional (3D) structures and all SnRK2-V proteins displayed the canonical Ser/Thr kinase fold. Each one revealed the well-ordered characteristic SnRK2 box, in which a single α-helix was found to be present in the N-terminal lobe packed parallel against the αC helix (Fig. 6). The C-terminal lobe was larger and mainly helical (Fig. 6). The activation loop was at amino acid positions 163–193 in SnRK2.8-V, 157–187 in SnRK2.9-V, and 158–188 in SnRK2.10-V protein. In the activation loop, nine amino acid sits were predicted to be the phosphorylation sites for SnRK2.8-V, SnRK2.9-V and SnRK2.10-V activation, respectively (Fig. 5). The analysis shown that the three proteins had the same phosphorylation sites. SnRK2.8-V included Y170, S171, S173, S174, S178, S182, T183, T186 and Y189. SnRK2.9-V contained Y164, S165, S167, S168, S172, S176, T177, T180 and Y183. While Y165, S166, S168, S169, S173, S177, T178,T181 and Y184 belonged to SnRK2.10-V.

### Cis-acting regulatory elements (CAREs) analysis of *SnRK2-V* genes

**Fig. 7:**
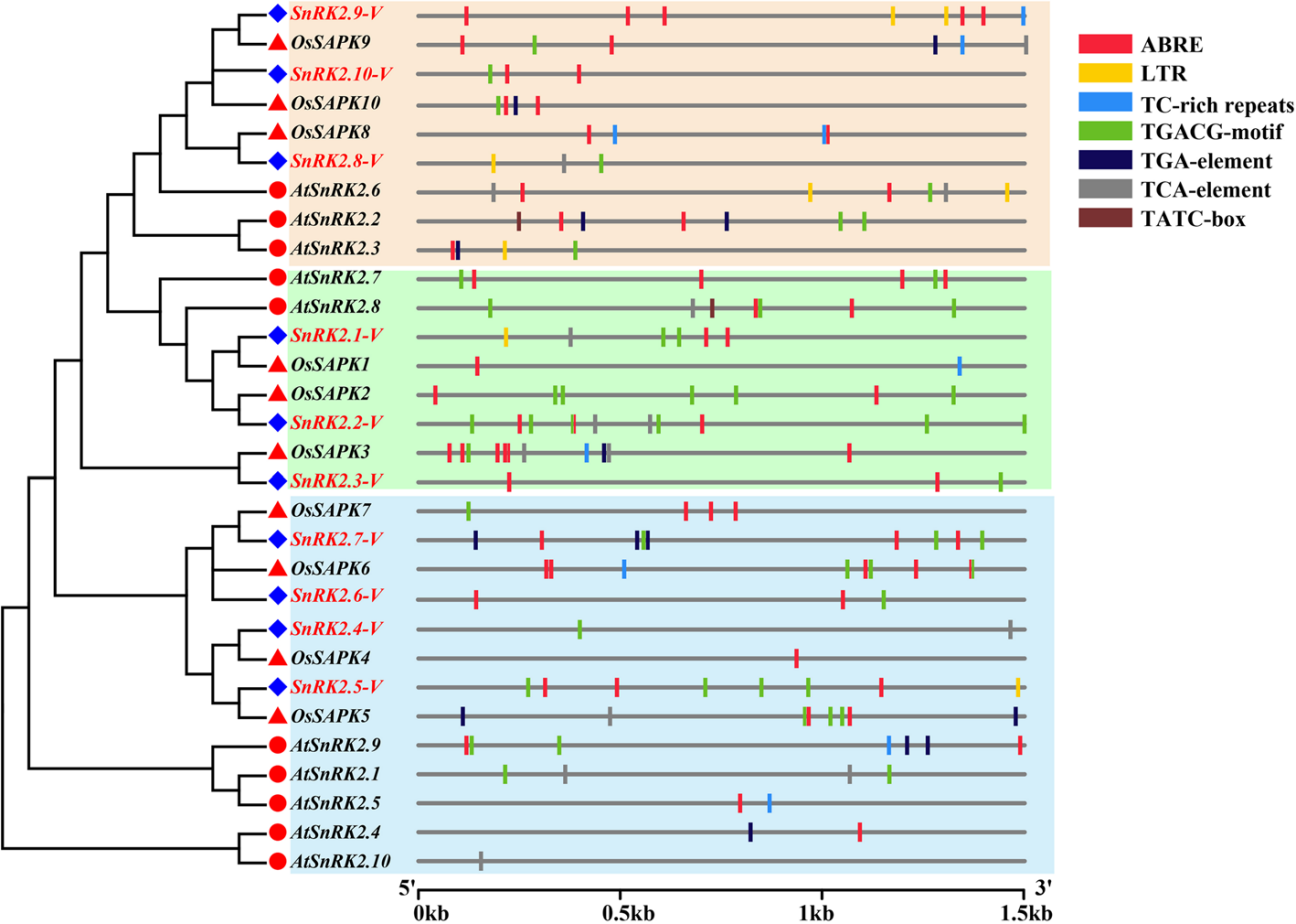
Cis-acting regulatory element (CAREs) locations of *SnRK2-V* genes. The 1.5 kb promoter sequences before the CDS of *SnRK2-V* genes were used to analyze hormone response CAREs, including ABRE, TGA-element, TATC-box, TGACG-motif, TCA-element, which responded to ABA, auxin (IAA), gibberellin (GA), methyl jasmonate (MeJA), salicylic acid (SA), as well as low-temperature-responsive (LTR) elements and defense/stress-responsiveness (TC-rich repeats). The black line represented the promoter length, other different color lines locating on the promoter represented the different cis-acting elements. Species abbreviations: *At*: *A. thaliana*; *Os*: *O. sativa*; *-V*: *H. villosa*

To better understand the potential function of *SnRK2-V* genes, the 1.5 kb gene sequences upstream of the CDS as the cis-acting regulatory elements of promoter were analyzed with the plant CAREs. Totally, four functional hormone response CAREs, including ABA, methyl jasmonate (MeJA), auxin (IAA), and salicylic acid (SA) response elements were obtained (Fig. 7; Table S2). Except *SnRK2.4-V* and *SnRK2.8-V* genes, other *SnRK2-V* genes had ABA-responsiveness (ABRE) and the maximum ABRE elements were five in *SnRK2.9-V* gene (Fig. 7). MeJA responsiveness (TGACG-motifs) was also enriched in most *SnRK2-V* genes. *SnRK2.2-V* had the largest MeJA responsiveness elements. Likewise, IAA and SA response elements were found in the *SnRK2-V* genes. Compared with SA-response elements (TCA-element) in *SnRK2.1-V*, *SnRK2.2-V*, *SnRK2.4-V* and *SnRK2.8-V*, the IAA-response elements found in *SnRK2-V* genes promoter only excited in *SnRK2.7-V*. However, none gibberellin (GA)-response elements (TATC-box) were detected in the *SnRK2-V* genes promoter sequences Additionally, the stress-related elements, including low-temperature-response (LTR) elements and defense/stress-response (TC-rich repeats) elements, were also identified in *SnRK2-V* genes. Except *SnRK2.9-V* containing two LTR elements, *SnRK2.1-V, SnRK2.5-V* and *SnRK2.8-V* only possessed one LTR in their promoter. More importantly, TC-rich repeats were present in only *SnRK2.9-V* promoter sequence*.*

### Subcellular localization and expression profiling of *SnRK2-V* genes

**Fig. 8:**
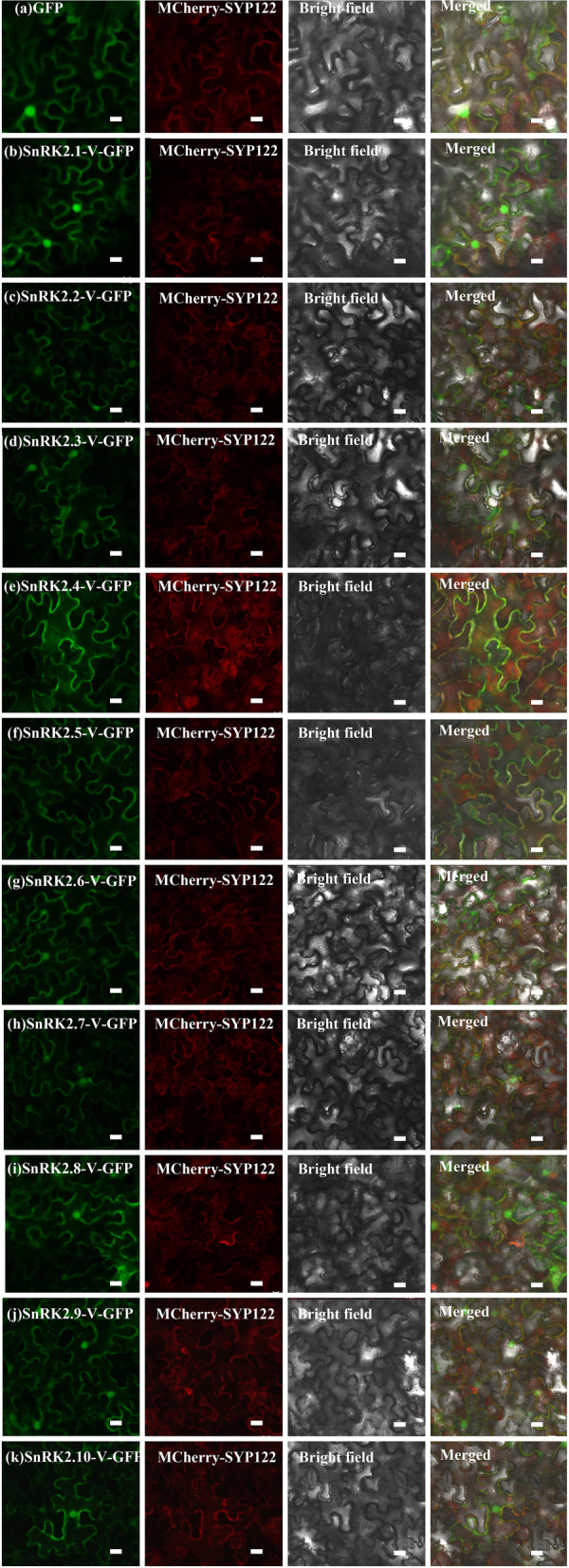
Subcellular localization of SnRK2-V in the epidermal cells of *Nicotiana benthamiana*: subcellular localization of GFP (**a**), SnRK2.1-V-GFP (**b**), SnRK2.2-V-GFP (**c**), SnRK2.3-V-GFP (**d**), SnRK2.4-V-GFP (**e**), SnRK2.5-V-GFP (**f**), SnRK2.6-V-GFP (**g**) SnRK2.7-V-GFP (**h**), SnRK2.8-V-GFP (**i**), SnRK2.9-V-GFP (**j**) and SnRK2.10-V-GFP (k). GFP was used as the control. The localization of mCherry-SYP122 was shown in red, and the localization of GFP and its fusion proteins were shown in green. Scale bar = 10 μm

Investigating the subcellular localization of a protein may provide clues towards the elucidation of its function. To verify where the SnRK2-V proteins were localized in *vivo*, the *Agrobacterium* method was performed to observe the transient expression of GFP (green fluorescent protein)-tagged fusion proteins in the leaves of *Nicotiana Benthamiana*. The GFP signals of the fusion proteins could be detected for all ten cloned SnRK2-V proteins, with the signals deferentially localized (Fig. 8). Compared with an even distribution of GFP fluorescence in the control group (Fig. 8a), all ten identified SnRK2-V proteins localized on the plasma membrane (PM) (Fig. 8). Notably, some SnRK2-V proteins also had obvious GFP fluorescence in the cytoplasm and nucleus, such as SnRK2.1-V, SnRK2.2-V, SnRK2.3-V, SnRK2.6-V, SnRK2.7-V, SnRK2.8-V, SnRK2.9-V and SnRK2.10-V (Fig. 8b, c, d, g, h, i, j, k).

To further predict the functions of the identified *SnRK2-V* genes*,* we investigated their transcription levels in different tissues or in responses to various biotic or abiotic stresses. The transcription patterns of *SnRK2-V* genes in different tissues of *H. villosa* (e.g., roots, stems, leaves, spikes, and grains) under two abiotic stresses (e.g., drought and salt) or two biotic stresses for *Blumeria graminis* f. sp. *Tritici* (*Bgt*) isolate E26 and *Puccinia sriiformis* f. sp. *Tritici* (*Pst*) isolate CYR32 were investigated by qRT-PCR. A three-fold change of transcription level was arbitrarily considered to be positive expression induction. Moreover, the tissue-specific expression analysis displayed that there were dramatic differences in the transcription levels of *SnRK2-V* genes. A relatively highest transcription level of *SnRK2.2-V* gene was detected in roots, *SnRK2.4-V* gene was mainly in stems and spikes, *SnRK2.9-V* gene was in leaves, and *SnRK2.8-V* gene was in grains (Figure S1). Meanwhile, the lowest transcription level of *SnRK2.3-V* gene was detected in grains, and the transcription level of *SnRK2.5-V* gene was in stems and grains. Contrarily, the lowest transcription level of *SnRK2.1-V* was detected in all tested tissues (Figure S1).

**Fig. 9:**
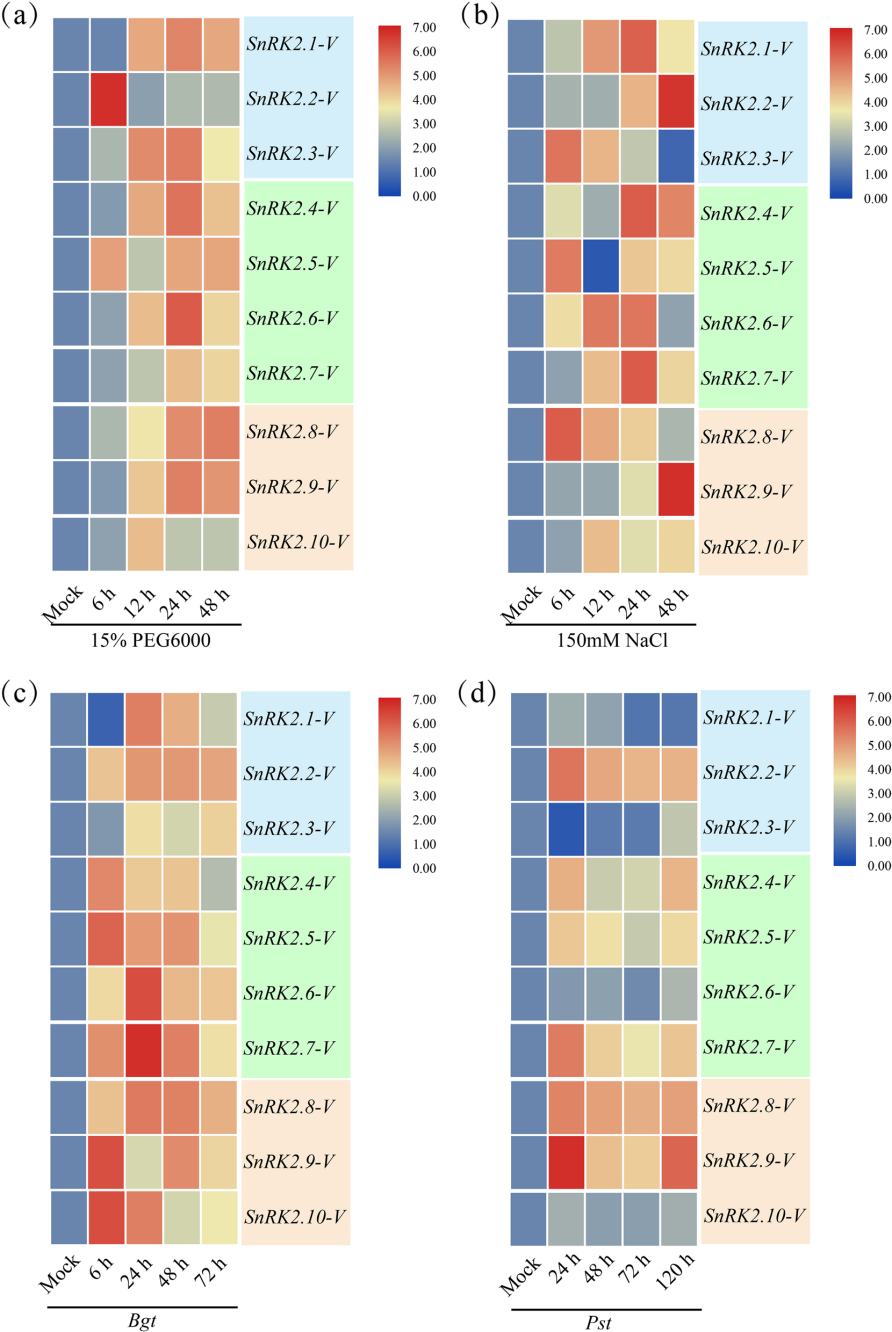
Transcription profiling of *SnRK2-V* genes after treatments with abiotic and biotic stresses. The transcription profiling of *SnRK2-V* genes in response to drought (**a**) and salt stress (**b**). The transcription profiling of *SnRK2-V* genes in response to *Bgt* (**c**) and *Pst* (**d**). The scale bar was showing transcription level of the genes. Abbreviations: *Bgt*: *Blumeria graminis* f. sp. *tritici*; *Pst*: *Puccinia sriiformis* f. sp. *tritici*

Under abiotic (e.g., drought and salt) or biotic (e.g., *Bgt* and *Pst*) stresses, different genes showed various expression patterns. Under drought stress, the transcription levels of *SnRK2.2-V* and *SnRK2.5-V* increased significantly at 6 h, and those in the other six *SnRK2-V* genes were up-regulated at 12 h, and increased to the maximum levels at 24 h (Fig. 9a). After NaCl treatment, transcription levels of four genes (e.g., *SnRK2.3-V*, *SnRK2.5-V*, *SnRK2.7-V* and *SnRK2.8-V*) were significantly up-regulated at 6 h, and those of *SnRK2.2-V*, *SnRK2.4-V* and *SnRK2.9-V* increased obviously at 24 h, and held high level up to 48 h (Fig. 9b). Of note, it was different from above *SnRK2-V* genes that the transcription levels of *SnRK2.1-V* and *SnRK2.7-V* were up-regulated at 12 h, and maintained till to 24 h (Fig. 9b). In response to *Bgt* and *Pst* infections, the transcription levels of six genes (e.g., *SnRK2.2-V*, *SnRK2.4-V*, *SnRK2.5-V*, *SnRK2.7-V*, *SnRK2.8-V* and *SnRK2.9-V*) were up-regulated at 6 h. Among them, the transcription level of *SnRK2.9-V* increased dramatically at 6 h, and that of *SnRK2.2-V*, *SnRK2.7-V* and *SnRK2.8-V* genes reached highest level at 24 h (Fig. 9c, d). Compared with *Pst* infection, the transcription levels of *SnRK2.1-V* and *SnRK2.3-V* genes were only up-regulated markedly under *Bgt* infection, increased significantly at 24 h, and reached the peak at 72 h (Fig. 9c).

To further verify the transcription levels of *SnRK2-V* genes induced by exogenous ABA, seedlings of *H. villosa* were transferred with ABA and then the samples were separately collected at 0, 6, 12, 24, and 48 h for qRT-PCR analysis. The data showed that, after ABA treatment, the transcript level of *SnRK2.8-V* gene was induced significantly at 6 h, and reached a maximum of five-fold change at 24 h. Compared with *SnRK2.8-V*, the transcription level of *SnRK2.9-V* increased obviously at 12 h, and peaked with a maximum of ten-fold change at 24 h (Figure S2). In contrary, the transcription levels of other *SnRK2-V* genes were not induced markedly by exogenous ABA treatment (Figure S2).

### Overexpression of *SnRK2.9-V* in wheat enhances drought and salt tolerances

**Fig. 10:**
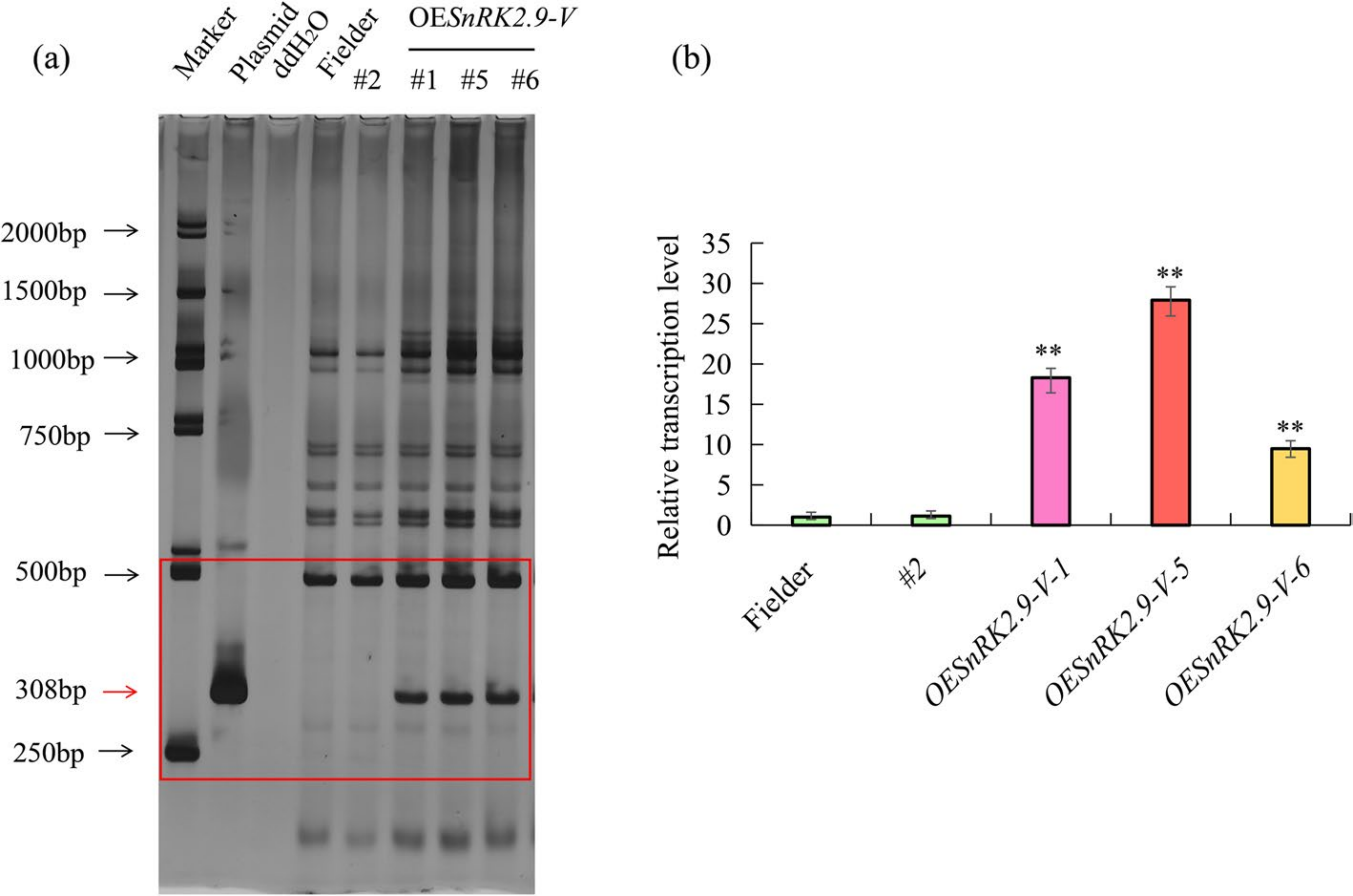
PCR identification and transcription profiling of *SnRK2.9-V* in common wheat transgenic plants. **a** PCR identification of *SnRK2.9-V* in common wheat transgenic plants. Lanes 1–8 represented Marker, plasmid, ddH_2_O, Fielder, negative control and three OE*SnRK2.9-V* transgenic wheat, respectively. The red arrow represented the target strip. **b** Related transcription levels of *SnRK2.9-V* in transgenic wheat. Significant differences are indicated as **P < 0.01, *P < 0.05. Data are mean ± SE (n ≥ 3)

Previous studies have demonstrated that SnRK2 proteins were involved in drought and salt stresses [6, 7]. To further confirm the positive function of *SnRK2.9-V* in drought and salt stresses, we generated three independent 2 × 35S:*SnRK2.9-V* transgenic OE lines (OE*SnRK2.9-V*#1, OE*SnRK2.9-V*#5 and OE*SnRK2.9-V*#6) in the wheat cv. Fielder, which carried the full CDS of *SnRK2.9-V*. The transgenic plants were confirmed by PCR using specific primers to amplify a 308 bp DNA segment in T_2_ generations. Compared with negative transgenic plant (#2) in which no amplification was detected (Fig. 10a). The results indicated that transcript levels of *SnRK2.9-V* in the leaves of three OE lines increased prominently compared with WT and #2 as was shown in Fig. 10b.

**Fig. 11:**
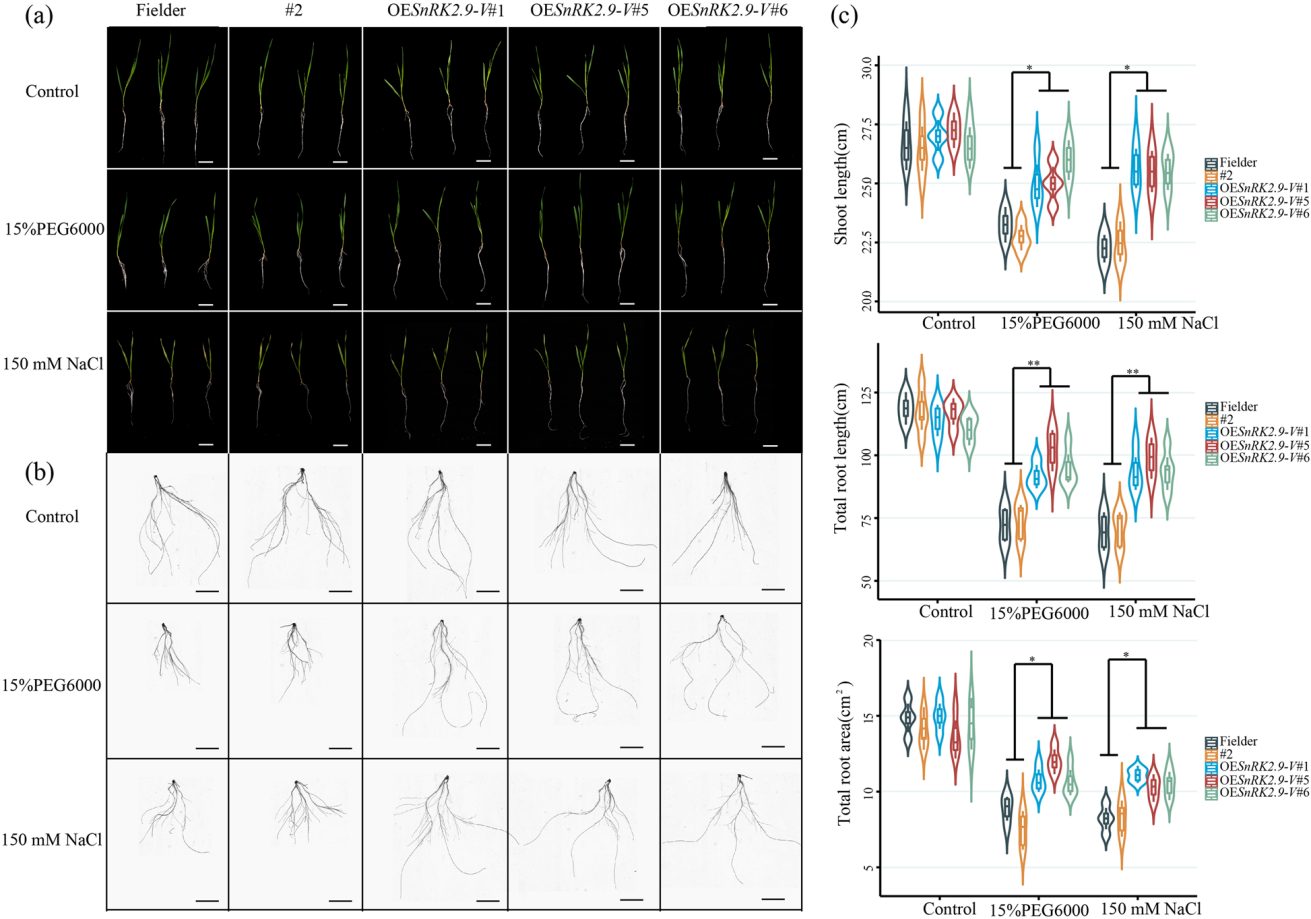
*SnRK2.9-V* overexpression improved drought and salt stresses in common wheat. **a** Growth performance of seeding stages under different treatments in control and OE*SnRK2.9-V*-T_2_ plants. Scale bar = 5 cm. **b** Scan analysis of root in seeding stages under different treatments in control and OE*SnRK2.9-V*-T_2_ plants. Scale bar = 5 cm. **c** Shoot length, total root length and total root area in seeding stages under different treatments in control and OE*SnRK2.9-V*-T_2_ plants. Significant differences are indicated as **P < 0.01, *P < 0.05. Data are mean ± SE (n ≥ 3)

To determine whether ectopic transcription of *SnRK2.9-V* gene influences their drought and salt tolerances, 14-days-old seedlings of positive transgenic lines (OE*SnRK2.9-V*#1, OE*SnRK2.9-V*#5 and OE*SnRK2.9-V*#6), Fielder and negative transgenic line (#2) were separately transferred to solution with 15% PEG6000 or 150 mM NaCl, and then compared with control (ddH_2_O) treatment. As shown in Fig. 11, the shoot lengths, total root lengths and total root areas displayed no visibly differences in OE*SnRK2.9-V* lines, Fielder and #2 under ddH_2_O condition. Conversely, under PEG6000 or NaCl treatments, the shoot and root growths of Fielder and negative line #2 were dramatically inhibited in comparison with positive transgenic lines (Fig. 11). After PEG6000 treatment, the shoot lengths of three positive lines decreased by 11.67%, 12.20% and 11.00%, respectively, in comparison with those with reduction of 14.20% in Fielder and 14.81% in #2 plants. Additionally, the total root lengths and total root areas of transgenic lines descended only 0.20-fold, with 21.00% and 20.20% in OE*SnRK2.9-V*#1, 18.25% and 20.67% in OE*SnRK2.9-V*#5, and 26.25% and 26.67% in OE*SnRK2.9-V*#6, respectively, in comparison with the two-fold reductions in Fielder with 46.25% and 46.67% and #2 plants with 43.10% and 48.00% (Fig. 11c). In response to NaCl treatments, the shoot lengths, total root lengths and total root areas decreased more than two-fold change in Fielder (20.93%, 43.84% and 43.89% respectively) and #2 negative line (21.00%, 40.67% and 40.89% respectively), showing a significantly enhanced tolerance in positive transgenic lines. As indicated in Fig. 11c, the shoot lengths, total root lengths and total root areas decreased much lower than those in control lines, in which positive transgenic lines reduced only by 11.67%, 22.25%, and 21.20% of OE*SnRK2.9-V*#1, 12.00%, 20.67%, and 22.25% of OE*SnRK2.9-V*#5, and 11.20%, 22.25%, and 22.25% of OE*SnRK2.9-V*#6, respectively.

### Overexpression of *SnRK2.9-V* enhances the transcription levels of antioxidase genes upon drought and salt stresses in wheat

**Fig. 12:**
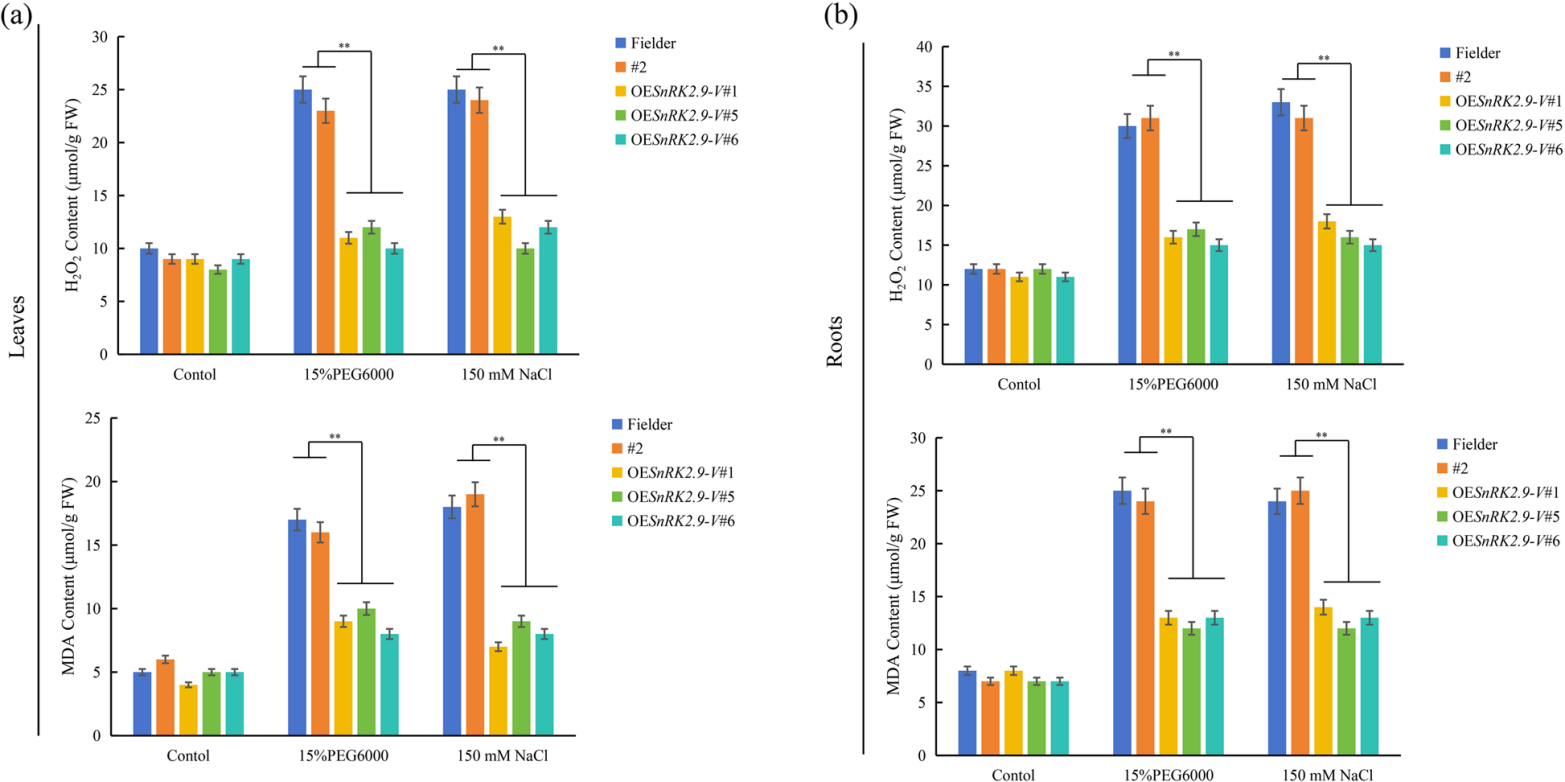
Physiological indices of the transgenic wheat plants overexpressing *SnRK2.9-V* under drought and salt stress. Two-week-old wheat plants grown on 1/2 MS medium were transferred to hydroponics for seven days with 1/2 MS medium supplemented with the 150 mM NaCl solution and15% PEG6000 solution. Leaves (**a**) and roots (**b**) of control and OE*SnRK2.9-V*-T_2_ plants under drought and salt treatments were sampled for the assessment of H_2_O_2_ and MDA. Significant differences are indicated as **P < 0.01, *P < 0.05. Data are mean ± SE (n ≥ 3)

Stresses are perpetually associated with the generation of ROS, such as H_2_O_2_, and ROS accumulation, leads to lipid peroxidation, and results in the production of MDA [20]. MDA is a stress-specific molecular marker that is indicative of the extent of membrane injury and cell and tissue damage. To determine whether overexpression of *SnRK2.9-V* can induce the ROS generation, we measured H_2_O_2_ and MDA contents in positive transgenic lines (OE*SnRK2.9-V*#1, OE*SnRK2.9-V*#5 and OE*SnRK2.9-V*#6), Fielder and negative transgenic line #2 under different conditions. Our data verified that H_2_O_2_ and MDA contents decreased in the positive transgenic lines under PEG6000 and NaCl conditions. In contrast, increases of H_2_O_2_ and MDA contents were observed in Fielder and negative transgenic line #2 after PEG6000 and NaCl treatment, indicating that overexpression of *SnRK2.9-V* can reduce oxidative damage via decreasing ROS content under PEG6000 and NaCl conditions (Fig. 12).

**Fig. 13:**
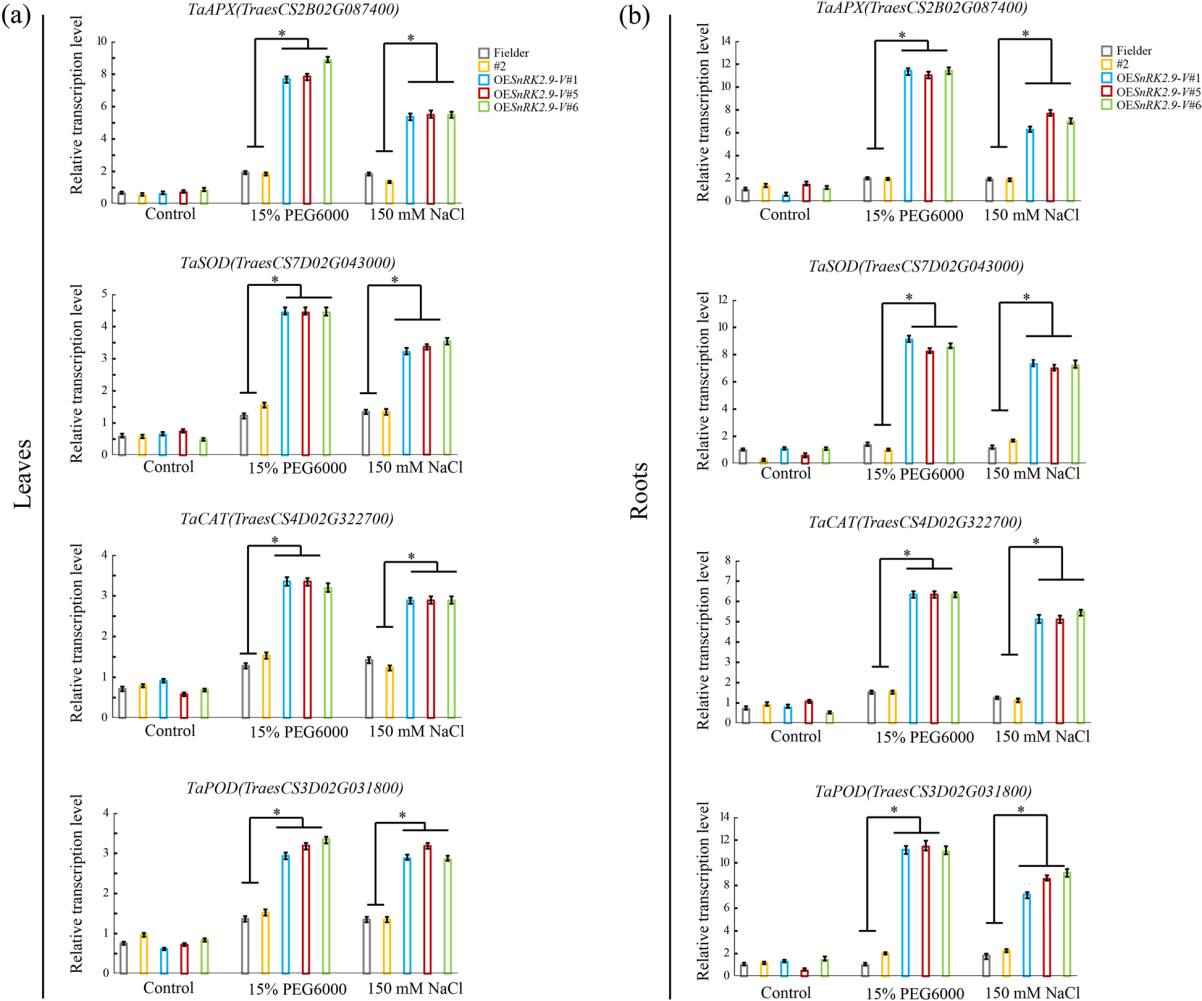
*SnRK2.9-V* regulates the transcription of antioxidant enzyme genes in transgenic wheat. **a** Transcription profiling of *TaAPX*, *TaSOD, TaCAT* and *TaPOD* in response to drought and salt treatments in the leaves of control and OE*SnRK2.9-V*-T_2_ plants. **b** Transcription profiling of *TaAPX*, *TaSOD, TaCAT* and *TaPOD* in the root of control and OE*SnRK2.9-V*-T_2_ plants under drought and salt treatments. Significant differences were indicated as **P < 0.01, *P < 0.05. Data were mean ± SE (n ≥ 3)

To elucidate the possible molecular mechanisms underlying the *SnRK2.9-V* gene in stress responses, the transcription levels of drought- and salt-responsive genes of antioxidant system including *TaAPX*, *TaSOD, TaCAT* and *TaPOD* in leaves (Fig. 13a) and roots (Fig. 13b) were separately investigated in positive transgenic lines, Fielder and negative transgenic line. A three-fold change of transcription level was arbitrarily considered to be represent positive expression induction. qRT-PCR analysis revealed no significant differences in the transcription levels of *TaAPX*, *TaSOD, TaCAT* and *TaPOD* genes between the positive lines and negative lines under normal conditions (ddH_2_O). Under PEG6000 and NaCl conditions, the elevated transcription levels of *TaSOD*, *TaCAT* and *TaPOD* were much higher in roots than those in leaves (Fig. 13)*.* The transcription levels of *TaSOD, TaCAT* and *TaPOD* genes increased 10.00-fold, 6.50-fold and 12.00-fold in the roots of OE*SnRK2.9-V* lines, and 4.50-fold, 3.50-fold and 3.50-fold in the corresponding leaves after PEG6000 treatment. However, the transcription levels of these genes were not markedly changed in the Fielder and #2 plants. In response to high salt condition, the transcription levels of *TaSOD, TaCAT* and *TaPOD* genes increased by 8.00-fold, 5.50-fold and 7.50-fold, respectively in the roots of OE*SnRK2.9-V* lines, along with the increased by 3.50-fold, 3.00-fold and 3.50-fold in the corresponding leaves. However, no significantly changes were detected in these transcription levels in Fielder and #2 plants as well as those under PEG6000 condition. Intriguingly, the maximal transcription level of *TaAPX* gene was detected in OE*SnRK2.9-V* lines*,* with 8.00-fold increase in the leaves compared with 6.00-fold in the roots under PEG6000 condition. Conversely, under high salt condition, the transcription level of *TaAPX* increased more than 5.00-fold in the leaves of OE*SnRK2.9-V* lines in comparison with 6.00-fold in the corresponding roots.

## Discussion

SnRK2 is a family of highly conserved protein kinases, which play important roles in plant adaptation to various environmental signals. With the completion of whole genome and high-quality genome assembly in *H. villosa*, understanding the whole gene family of *SnRK2-V* is helpful for researchers to explore the functions of specific *SnRK2-V* genes in various stress responses.

### Evolutionary feature of *SnRK2* gene family

In plants, the *SnRK2* gene family has been well investigated. Until now, 108 *SnRK2* genes have been isolated from plants*,* including ten *SnRK2* genes in *A. thaliana* [8], ten in *O. sativa* [9], thirty in *T. aestivum* [16], eight in *T. urartu*, ten in *Ae. speltoides*, ten in *Ae. tauschii*, twenty in *T. dicoccoides*, and ten in *H. vulgare* [15]. Our study revealed that there were ten *SnRK2-V* genes in *H. villosa*, a diploid wild relative of wheat. It was similar to rice, Arabidopsis and other diploid Triticeae species, in which seven chromosome pairs possessed ten *SnRK2* genes. All these data indicate that the *SnRK2-V* gene family is evolutionarily conserved in Triticeae species in the context of gene number [15, 16]. The widely accepted classification system helps to categorize structures of *SnRK2-V* genes into three subgroups (three in Group I, four in Group II and three in Group III), which are well defined in wheat and other plants, as well as similar members proportion of each subgroup between *H. villosa* and other diploid species, suggesting that the classification of *SnRK2-V* genes is also conserved. Moreover, analysis of chromosome location indicated that the *SnRK2-V* genes were distributed on the chromosome 1, 2, 3, 4 and 5. This indicates that *SnRK2-V* genes were formed before the differentiation of Triticeae species [36–39].

### *SnRK2-V* family members undertake diverse roles

The complete genome sequences for conserved domains, cis-elements and gene expressions in *H. villosa* offer an opportunity to learn more about functional characterization of the individual *SnRK2-V* genes. At present study, ten *SnRK2-V* genes contained highly conserved domains at the N-terminal kinase domains regions with an ATP binding site, alpha C helix, the serine/threonine protein kinase active-site and activation loop, which is highly consistent with rice and Arabidopsis [8, 9]. These demonstrated that the N-terminal kinase of *SnRK2-V* genes was evolutionarily conserved in Triticeae species [15, 16]. Otherwise, domain II with osmotic stress-mediated activation existed in all the promotor regions of *SnRK2-V* genes, while only *SnRK2.8-V*, *SnRK2.9-V* and *SnRK2.10-V* existed ABA-responsive kinases in the domain II. Accumulating evidence has demonstrated that, under exogenous ABA treatment, the transcription level of *SnRK2.9-V* increased obviously by ten-fold, with five markedly induced *SnRK2* genes (*AtSnRK2.2*, *AtSnRK2.3, AtSnRK2.6*, *AtSnRK2.7* and *AtSnRK2.8*) in Arabidopsis [8], strongly induced Os*SAPK8*-Os*SAPK10* in rice [9] and prominently induced *TaSnRK2.8*-*TaSnRK2.10* in Wheat [16]. Additionally, the most frequently present types of CAREs were ABREs, and TGACG-motifs, TCA-elements and LTRs, which are separately present in the promoters of *SnRK2.4, SnRK2.6* and *SnRK2.8* genes [40]. Conversely, *SnRK2.9-V* had the maximum ABREs numbers, and TC-rich repeats were only located in *SnRK2.9-V.* Moreover, tissue-specific expression analysis displayed the different transcription patterns of *SnRK2* genes. These evidences show that different members of *SnRK2-V* family have different functions [41]. However, their specific functions need to be further validated by transgenic approach.

Expression pattern analysis can provide an opportunity to estimation of gene functions, and the transcription levels of *SnRK2* genes can be obviously induced in various types of abiotic stresses [42]. Among them, *AtSnRK2.4* and *AtSnRK2.10* were strongly induced under salt treatment [28]. Under high salt and PEG conditions, rice *OsSAPK1* and *OsSAPK2* genes [30] and potato (*Solanum tuberosum*) *StSnRK2.4* gene [43] were rapidly activated. Similarly, *GhSnRK2.3/2.7/2.8/2.9/2.10* genes in cotton (*Gossypium hirsutum*) were notably induced under salt and PEG conditions [44], and *TaSnRK2.4*, *TaSnRK2.7*, and *TaSnRK2.8* genes in wheat were up-regulated in response to drought and high salt [16]. In our current study, we analyzed the expression profiles of *SnRK2-V* genes under abiotic (drought and salt) stress and verified that ten *SnRK2-V* genes were shown to respond to drought and salt stresses, emphasizing that these genes may be positive regulators in responses to drought and salt stresses. Simultaneously, we also detected the transcription levels of *SnRK2-V* genes under biotic (*Bgt* and *Pst*) stress and demonstrated that *SnRK2.1-V* and *SnRK2.3-V* did not respond to *Bgt* infection. Notably, six *SnRK2-V* genes including *SnRK2.2-V*, *SnRK2.4-V*, *SnRK2.5-V*, *SnRK2.7-V*, *SnRK2.8-V* and *SnRK2.9-V* were dramatically induced by *Bgt* and *Pst* infection. Therefore, these six *SnRK2-V* genes are worthy to be further studied for their function in disease resistance for the demonstrated cross-link between abiotic and biotic stress regulation [45, 46].

### *SnRK2.9-V* acts as the positive regulator of drought and salt tolerances via antioxidant system

Several studies have demonstrated the positive roles of *SnRK2* genes in numerous defenses against harsh environments (e.g., drought and salinity) [46–50]. Overexpression of *SnRK2* member *OsSAPK4* or *OsSAPK6* improved SnRK2 transgenic rice drought tolerance [51], and overexpression of *B. distachyon SnRK2.9* dramatically improved drought and high salt resistance [12]. Besides, heterologous overexpressing *TaSnRK2.9* in *N. tabacum* significantly enhanced tolerances of transgenic plants to drought and salt stresses [24], and heterologous overexpressing *TaSnRK2.4* in Arabidopsis improved salt resistance [16]. Similarly, heterologous overexpressing *TaSnRK2.3* in Arabidopsis enhanced tolerance to drought by more developed roots [49]. In our study, *SnRK2.8-V*, *SnRK2.9-V* and *SnRK2.10-V* showed sensible responses to drought and high salt condition. It closely resembled the results from the orthologous genes *AtSnRK2.2*, *AtSnRK2.3* and *AtSnRK2.6* in regulating ABA synthesis and relevant defense-signaling under drought [8]. However, defense/stress-responsiveness (TC-rich repeats) only existed in the promoter sequence of *SnRK2.9-V,* indicating a conservative function of *SnRK2.9-V* in stress resistance. Consistently, our research demonstrated that there were significantly enhanced tolerance to drought and high salt in *SnRK2.9-V* transgenic lines, implying that ectopic expression of *SnRK2.9-V* can improve the tolerance of wheat to drought and salt stresses. Collectively, our present study and others indicate that *SnRK2.9-V* may represent a gene resource for improving wheat resistance to drought and soil salinization stresses.

To survive biotic and abiotic stresses, plants have developed elaborate mechanisms to adaptation by modulating the expression of genes. As we know, the most important functions of SnRK2 proteins against abiotic stresses are their phosphorylation modifications. AtSnRK2.6 phosphorylated CHYR1 (E3 ubiquitin ligase) and bZIP transcription factors ABF1/2/3/4 to enhance drought stress tolerance [52], and it also phosphorylated the transcription factor ICE1, activated the transcription of *CBF* genes, and enhanced low-temperature tolerance [53]. Additionally, overexpression of rice *OsSAPK6* enhanced phosphorylation level of OsABF transcription factors and increased salt tolerance [54]. Moreover, OsSAPK9 positively enhanced drought stress through the potentiality of transactivation of the OsbZIP transcription factors [20]. ZmSnRK2.11 effectively alleviated the damage caused by high salt intake and drought stresses via phosphorylating ZmABI1/ABI2/DREB2A/P5CS1 [55]. In response to drought, αC-helix in the N-terminal lobe is required for correct folding of the catalytic centre and kinase activation loop, which is stabilized by the SnRK2 box [42]. In our search, ten SnRK2-Vs were highly conserved at the N-terminal containing the serine/threonine protein kinase active-site and activation loop, but their C-terminal varied significantly, therefore their features are identical to other Snf1 kinase domains [20]. They also displayed the well-ordered characteristic SnRK2 box with a single α-helix in the N-terminal lobe packed parallel against the αC helix. In the activation loop, the predicted phosphorylation site S176 and T177 of SnRK2.9-V corresponded to the same positions of OsSAPK9, which had both autophosphorylation and transphosphorylation activities in vitro [20]. Taken together, SnRK2.9-V has a characteristic kinase fold structure and the recombinant protein might possess autophosphorylation and transphosphorylation activities. However, the phosphorylation function of SnRK2.9-V requires more biochemical experiments to further verify.

It is well known that APX, SOD, CAT and POD are key enzymes that play a decisive role in scavenging ROS and relieving oxidative stress to keep normal growth and development of plant [56, 57], and corresponding genes have been reported to be up-regulated upon various stresses [27, 58–61]. Several studies have demonstrated that overexpression of *SnRK2*s can promote an increase in activities or transcription levels of antioxidant enzymes, such as SOD, APX, CAT and POD, to scavenge ROS and maintain ROS homeostasis, thus relieving oxidative stress to keep the normal growth and development of plants under osmotic stresses [41, 45, 46]. In our research, heterologous overexpression of *SnRK2.9-V* increased the transcriptions of ROS scavenging enzyme-related genes, such as *TaAPX*, *TaSOD, TaCAT* and *TaPOD* in leaves and roots under high salt and drought stresses. Conversely, the orthologous gene *TaSnRK2.9* also activated SOD, CAT, and POD antioxidant system genes to reduce the H_2_O_2_ accumulation in transgenic tobacco under drought or salt stresses [24]. Likewise, its orthologous gene *BdSnRK2.9* transgenic plants exhibited lower levels of H_2_O_2_ under drought and high salt conditions [12]. Moreover, overexpressing *SAPK9* in planta enhanced the drought tolerance phenotype of transgenic lines through ROS detoxification, consequently reducing membrane damage [20]. Besides, overexpression of *CsSnRK2.5* improved its drought resistance through reducing the accumulation of ROS [32]. Our results of *SnRK2.9-V* supported its involvement in drought and salt stresses through expanding the transcription levels of antioxidant enzymes genes. Nevertheless, more biochemical experiments are needed to confirm the role of *SnRK2.9-V* in regulating antioxidant enzymes genes directly.

## Conclusions

Collectively, our study provides comprehensive insights into the *SnRK2* gene family in *H. villosa*. Classification of all *SnRK2-V* genes from the whole genome survey further contributes to the fundamental researches for the better understanding of key *SnRK2-V* genes against various biotic and abiotic stresses. Meanwhile, the preliminary verification of *SnRK2.9-V* in enhancing common wheat resistance to drought and salt stresses might provide a useful gene resource for improving wheat resistance in breeding.

## Materials and methods

### Plant materials

*H. villosa* (genome VV, accession no. 91C43) was obtained from Cambridge Botanical Garden in the UK and maintained by the Cytogenetic Institute, Nanjing Agricultural University (CINAU) in the 1970s [36]. Sun et al. used cytogenetics methods to identify *H.villosa* in our laboratory in 2018 [62]. Zhang et al. used this plant material for genome sequencing in our laboratory in 2023 [36]. And this plant material was used for gene cloning and expression analysis in this study [63]. Wheat varieties Fielder was maintained in Cytogenetics Institute of Nanjing Agricultural University (CINAU) and it was performed as described by Fan et al. [64]. And Fielder was used as the recipient cultivar for genetic transformation. *N. benthamiana* (NC89) plants were maintained in Cytogenetics Institute of Nanjing Agricultural University (CINAU) and it was performed as described by Zhang et al. [65]. And it used for subcellular localization analysis. All plants were grown in the greenhouse and the growth conditions were as follows: 14/10 h day/night cycle, 24/20 °C day/night temperature, 8,000 lx light intensity, and 70% relative humidity [63, 65].

### Identification of *SnRK2* gene family in Triticeae

The identified SnRK2s protein sequences of *A. thaliana* (*At*) and *O. sativa* (*Os*) were used as query sequences to blast (E-value ≤ 10^−10^) against protein database of the other species, including *T. urartu* (AA, 2n = 2x = 14) (http://plants.ensembl.org/index.html) [66], *T. aestivum* (AABBDD, 2n = 6x = 42) (https://www.ncbi.nlm.nih.gov) [67], *Ae. speltoides* (BB, 2n = 2x = 14) (http://202.194.139.32/expression/index.html) [68], *T. dicoccoides* (AABB, 2n = 4x = 28) (https://www.ncbi.nlm.nih.gov) [69], *Ae. tauschii* (DD, 2n = 2x = 14) (http://wheatomics.sdau.edu.cn/download.html) [70], *H. vulgare* (HH, 2n = 2x = 14) (http://plants.ensembl.org/index.html) [71] and *H. villosa* (VV, 2n = 2x = 14) [36]. After removing the redundant gene sequences for each species, the alignment hits were validated by performing a CD search as described above.

### Phylogenetic analysis and gene characteristics of *SnRK2* gene family in Triticeae

Multiple sequence alignment was conducted by ClustalW which was integrated in MEGA 7.0 [72]. Phylogenetic analysis was performed through online software PhyML 3.0 [73] using maximum-likelihood method with default parameter [74]. To understand the phylogenetic relationship of *SnRK2* genes, the unrooted phylogenetic tree was built using MEGA 7.0 via the Neighbor Joining (NJ) method.

Protein properties of *SnRK2* genes, including the relative molecular weight (MW) and isoelectric point (PI), were predicted using ExPASy (http://www.expasy.org/). WoLF PSORT (http://www.genscript.com/wolf-psort.html) was used for *SnRK2* gene family member subcellular localization predictions [63]. A multiple sequence alignment of the amino acid sequences of *SnRK2* genes in selected plant genomes was generated using DNAMAN (ver. 6.0) with default settings [63]. Crystal structure analysis of SnRK2s were done using Phyre2 web tools (http://www.sbg.bio.ic.ac.uk/phyre2/html) and EzMol 2.1 (http://www.sbg.bio.ic.ac.uk/~ezmol/) [63]. And phosphorylation sites were predicted using NetPhos 3.1 Server (http://www.cbs.dtu.dk/services/NetPhos).

### Chromosomal distribution and exon–intron structure of *SnRK2* gene family in Triticeae

Chromosomal information of predicted *SnRK2* genes was obtained from each species and their chromosomal locations were determined after using cDNA sequences as the query sequences blasted to the genomic sequences [63, 65]. Then we drew their locations onto the physical map of each chromosome using MapInspect tool (http://mapinspect.software.informer.com/) [63, 65]. MCScanX tool kit was used to investigate gene duplication events within species and sequence similarity between *SnRK2* genes in wheat and other plant species.

The gff3 files of each species were downloaded from the Ensembl Plants FTP server (http://plants.ensembl.org/index.html) for exon–intron structure analysis, and *SnRK2* genes structures were analyzed using the Gene Structure Display Server (GSDS) program (http://gsds.cbi.pku.edu.cn/) [63].

### Cis-acting regulatory elements (CAREs) analysis

To analyze putative cis-acting elements in the promoter region, 1.5-kb promoter regions were selected and screened against the Plant CARE database (http://bioinformatics.psb.ugent.be/webtools/plantcare/ html) [15]. Thereafter, the number of occurrences for each CARE motif was counted for *SnRK2* genes, and the most commonly occurring CAREs were used to produce figures in TBtools.

### Cloning of *SnRK2-V* genes from *H. villosa*

Prediction of *SnRK2-V* genes in *H. villosa* was carried out by comparing the genomic data of *H. villosa* [36], and the predicted proteins were processed according to the above method. According to the sequences, the primers (Table S3) were designed to clone the full-length cDNA of *SnRK2-V* genes from *H. villosa* using online software Primer3 (v. 0.4.0, University of California, Oakland, CA, USA) [65]. cDNA of *H. villosa* tissues (root, stem, leaf and grain) was served as a template for the isolation, and the specific primers for *SnRK2-V* genes of *H. villosa* were used for cloning [63]. This was performed at 95 °C for 30 s, followed by 32 cycles of 95 °C for 30 s, 56 °C for 45 s, and 72 °C for 1 min, and then 5 min at 72 °C in Phanta Max Super-Fidelity DNA polymerase (Vazyme, Nanjing, China) [63].

### Plant treatments

Plant treatments were performed as described by Zhao et al. [63] and Zhang et al. [75] with some modifications. Sterilized seeds were germinated and cultured with water in a climate chamber under a 12 h light/12 h dark cycle at 22 °C. The seedlings of *H. villosa* were grown in aqueous solution with 1/2 Murashige and Skoog (MS) medium or soil [63, 75]. Three-leaf stage *H. villosa* seedlings were in a plethora of multiple abiotic and biotic stresses. For *Bgt* infection, the plants were inoculated with *Bgt* virulent race E26 and the leaf tissues were sampled at 0, 6, 24, 48 and 72 h after inoculation [63, 75]. For *Pst* infection, the plants were inoculated with *Pst* virulent race CYR32 and the leaf tissues were sampled at 0, 24, 48, 72 and 120 h after inoculation. For drought stress, the plants were transferred to 1/2 MS medium with 15% PEG 6000, all leaf tissues were collected 0, 6, 12, 24 and 48 h [63, 75] For high salinity treatment, the plants were transferred to 1/2 MS medium with 150 mM NaCl, all leaf tissues were collected 0, 6, 12, 24 and 48 h [63, 75]. For phytohormones treatments, the plants were sprayed with 0.2 mmol abscisic acid and all leaf tissues were collected at 0, 6, 12, 24 and 48 h after spraying. All the samples were rapidly frozen in liquid nitrogen, then stored in an ultra-freezer (-80 °C) before use [63, 75].

For the analysis of high salinity and drought tolerances, seeds of *SnRK2.9-V* transgenic plants and the receptor variety Fielder were sterilized with 75% ethanol for 10 min and 12% sodium hypochlorite for 10 min respectively, and then washed five times with sterile water [65]. Wild type and *SnRK2.9-V* transgenic plants were cultured in 1/2 MS medium for two weeks. For the high salt and drought stresses, seedlings at 14 days old stage were cultured with hydroponics for seven days with 1/2 MS medium supplemented with the 150 mM NaCl solution and 15% PEG6000 solution. Therefore, the shoot lengths, total root lengths and total root areas of transgenic lines and the receptor variety were measured and analyzed. At the one-week drought and salt stress treatment stage, leaves and roots from each line were collected to measure. All the samples were rapidly frozen in liquid nitrogen, then stored in an ultra-freezer (-80 °C) before use. The averages of data were computed based on the means of three independent experiments, and each based on at least 10 independent plants [65].

### RNA isolation and transcription analysis of ROS-related genes

Total RNA was extracted using a Trizol Reagent kit (Invitrogen, CA, USA) according to the manufacturers’ instructions [63]. Three microliters RNA was used in agarose gel electrophoresis to check the quality and integrity of the obtained RNA samples. The first-strand cDNA was synthesized with random oligonucleotides using the HiScript® II Reverse Transcriptase system (Vazyme, Nanjing, China).

The transcription levels of *TaCAT* (*TraesCS4D02G322700*)*, TaAPX* (*TraesCS2B02G087400*)*, TaSOD* (*TraesCS7D02G043000*) and *TaPOD* (*TraesCS3D02G031800*) were analyzed by qRT-PCR. Tubulin was used as the internal control for normalization. Primers used for qRT-PCR were designed by Primer3 (Table S3), and three biological replications were performed. qRT-PCR was carried out in a total volume of 20 μL containing 2 μL of cDNA, 0.4 μL gene-specific primers (10 μM), 10 μL SYBR Green Mix, and 7.2 μL RNase free ddH_2_O, using the Roche LightCycler480 Real-time System (Roche, Basel, Swiss Confederation) [63]. Finally, the transcription lervel was represented in the form of relative fold change using the 2^−ΔΔCT^ method [76].

### Determination of H2O2 contents

Determination of H_2_O_2_ contents were performed according to the method of Malondialdehyde Microplate Assay Kit (Absin, Shanghai, China). Briefly, wild type and *SnRK2.9-V* transgenic plants were cultured in 1/2 MS medium for two weeks. For the high salt and drought treatments, seedlings at 14-day old stage were cultured with hydroponics supplemented with 150 mM NaCl solution or 15% PEG6000 solution for seven days. Then, 0.1 g fresh tissue of leaves or roots were weighed and 1 mL pre-cooled acetone was added for ice bath homogenization. The sample was transferred to an EP tube and diluted to 1 mL with acetone for centrifugation at 12,000 rpm at 4 °C for 10 min. Thereafter, 0.5 mL supernatant was taken and mixed well with Substrate and Reaction buffer on ice for centrifugation at 12,000 rpm at 25 °C for 10 min, the supernatant was removed and mixed with Dissolution Buffer well. Finally, 200 μL reaction liquid was added into a microplate to measure the absorbance at 415 nm. H_2_O_2_ content (μmol/g) = 100 × (OD_Sample_-OD_Black_)/(OD_Standard_-OD_Black_)/W.

### Determination of malondialdehyde (MDA) contents

MDA contents were measured according to the method of Hydrogen Peroxide Microplate Assay Kit (Absin, Shanghai, China). Wild type and *SnRK2.9-V* transgenic plants and seedlings with high salt and drought stresses were cultured as above mentioned. Then 0.1 g fresh tissue of leaves or rootswere taken and 1 mL Assay Buffer was added for ice bath homogenization. After centrifugation at 8,000 g at 4 °C for 10 min, the supernatant was taken and placed on ice for detection. Briefly, 10 μL sample was added into a 1.5 mL centrifuge tube and mixed with 0.6 mL Reaction Buffer (100 μL Reaction Buffer I and 10 μL Reaction Buffer II) gently. After keeping warm in a 90 °C-water bath for 30 min (cover tightly to prevent moisture loss), the tube was cooled in an ice bath and then centrifuged at 10,000 g for 10 min at 25 °C. Thereafter, 200 μL reaction liquid was added into a microplate to measure the absorbance at 532 nm. MDA content (μmoL /g) = (OD_Sample_-OD_Black_)/(OD_Standard_-OD_Black_)/W.

### Subcellular localization assay

Subcellular localization was performed as described by Zhao [63] with the following modifications. The ORFs of *SnRK2-V* genes (without stop codon) were amplified from the pTOPO-Blunt Vector, then inserted into the *p*Cambia1305-GFP vector, which contains the green fluorescent protein (GFP) reporter gene driven by the CaMV 35S promoter, using homologous cloning technology as per the manufacturers’ instructions (Vazyme, Nanjing, China) (Table S3). The 1305-GFP fusion constructed plasmid was transformed into *N. benthamiana* epidermal cells by *Agrobacterium tumefaciens* (strain GV3101) bacteria and incubated in darkness at 22 °C for 48 h. Then, the fluorescence signals were observed under confocal microscopy (LSM780, Zeiss, Oberkochen, Germany) according to the methods described by Wang and Zhang [76, 77].

### Genetic transformation

*SnRK2.9-V* was cloned into the plant expression vector *p*WM110 (driven by the CAMV 35S promoter) to generate vector *p*WM110:*SnRK2.9-V*. Then, the vector *p*WM110:*SnRK2.9-V* was transformed by *A. tumefaciens* to young embryos of wheat cultivar Fielder [65]. Regenerated plants were transplanted in the greenhouse and used for further characterization [63], and they were analyzed via PCR using conjugated gene-specific primers (Table S3) to identify positive transgenic plants.

### Statistical analysis

All statistical analyses were performed using SPSS version 13.0 (SPSS, Chicago, IL, USA). Data are presented as means and standard error (SE), and analyzed by a student’s t-test to check for quantitative differences between two different treatments [77]. *P* < 0.05 was set as the significance cut-off.

### Supplementary Information

**Supplementary Material 1.**

**Supplementary Material 2.**

## Data Availability

The protein data and genomic data used for SnRK2 identification from different species were provided in the Table S1, which were obtained from Ensembl Plants database, including: *Triticum urartu* (http://plants.ensembl.org/index.html), *Triticum aestivum* (https://www.ncbi.nlm.nih.gov), *Aegilops Speltoides* (http://202.194.139.32/expression/index.html), *Triticum dicoccoides* (https://www.ncbi.nlm.nih.gov), *Aegilops tauschii* (http://wheatomics.sdau.edu.cn/download.html) and *Hordeum vulgare* (http://plants.ensembl.org/index.html).

## Abbreviations

ABA: Abscisic acid
APX: Ascorbate peroxidase
ATP: Adenosine triphosphate
bp: Base pair
CAT: Catalase
CDS: Coding DNA sequence
DNA: Deoxyribonucleic acid
GA: Gibberellin
GFP: Green fluorescent protein
IAA: Auxin
KDa: Kilodalton
MDA: Malondialdehyde
MeJA: Methyl jasmonate
POD: Peroxidase
qRT-PCR: Quantitative real time polymerase chain reaction
RNA: Ribonucleic acid
ROS: Reactive oxygen species
SA: Salicylic acid
SnRK2: Sucrose nonfermenting-1-related protein kinase 2
SOD: Superoxide dismutase
