# Supplementary material for: Characterization of sucrose nonfermenting-1-related protein kinase 2 (SnRK2) gene family in Haynaldia villosa demonstrated SnRK2.9-V enhances drought and salt stress tolerance of common wheat
Source: BMC Genomics. 2024 Feb 26;25:209. doi: 10.1186/s12864-024-10114-7 (PMC10895793; doi:10.1186/s12864-024-10114-7)
Supplement: Supplementary file 1 — Supplementary Material 1. [file 12864_2024_10114_MOESM1_ESM.doc]

**Supplementary Information**


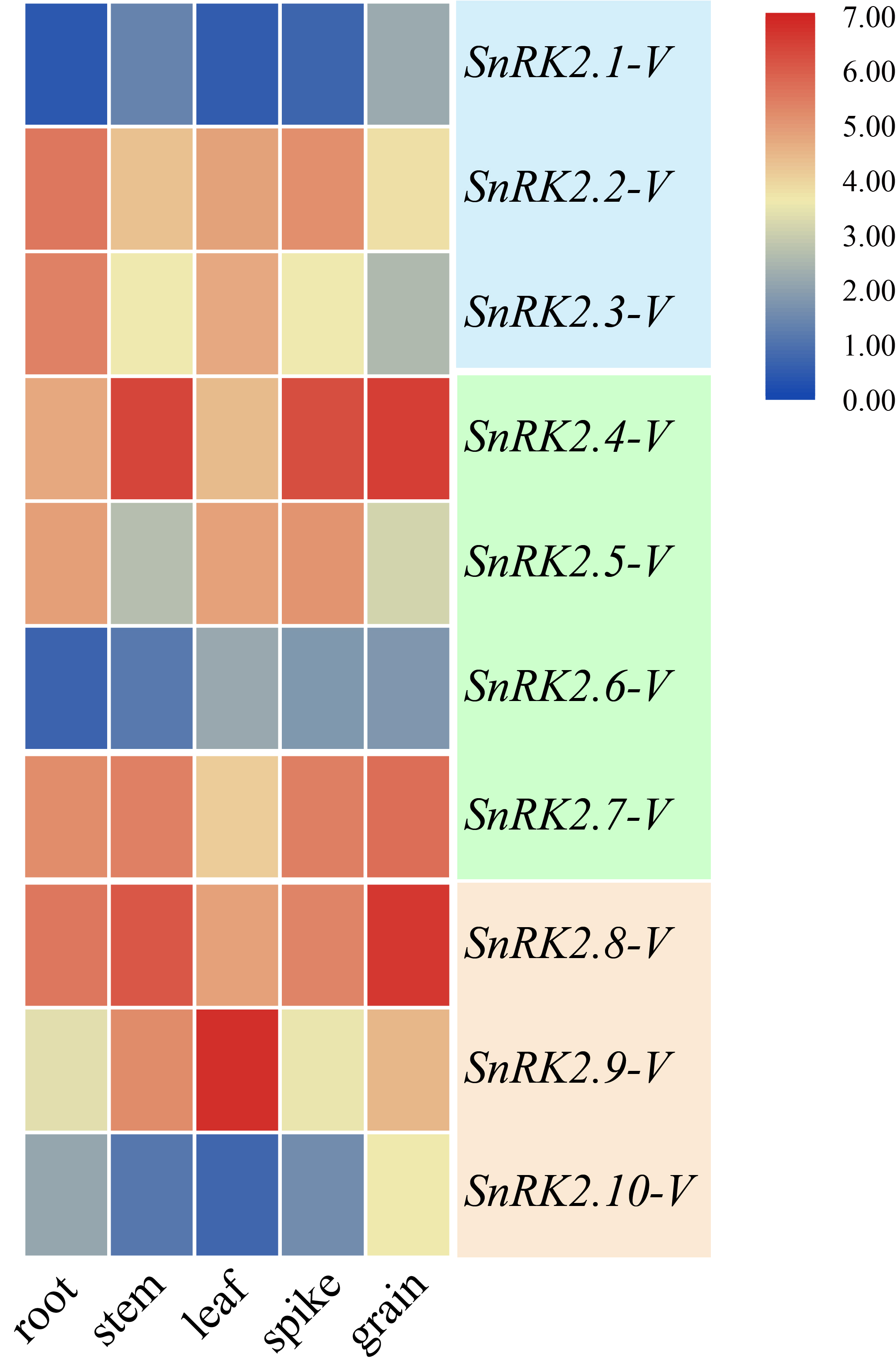


**Figure S1.** The transcription profiling of *SnRK2-V* genes in different tissues. The scale bar was showing transcription level of the genes.


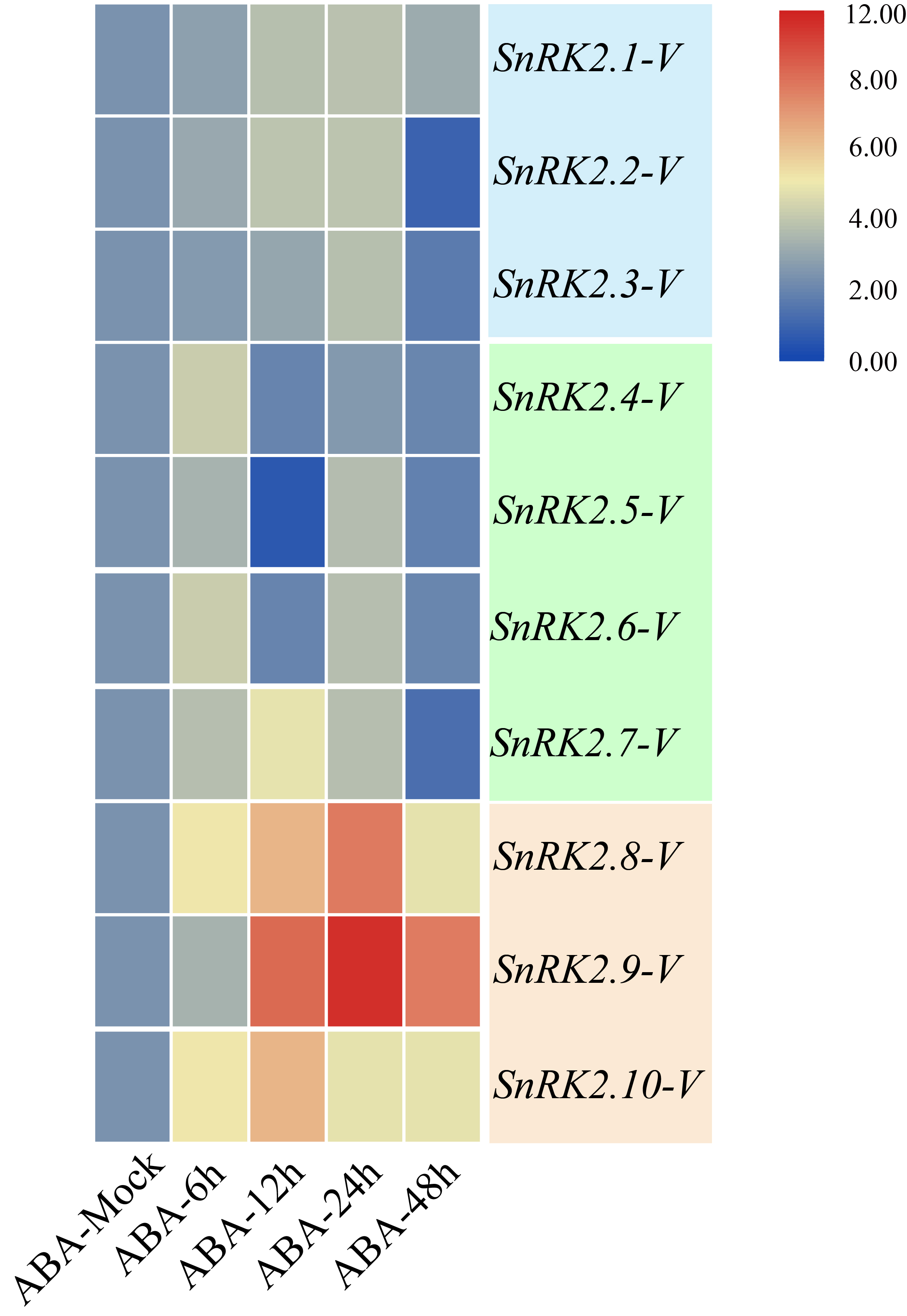


Figure S2. Heatmap of the transcription profiling of *SnRK2-V* genes in response to exogenous ABA treatment. The scale bar was showing transcription level of the genes.
