# Supplementary material for: Characterization of sucrose nonfermenting-1-related protein kinase 2 (SnRK2) gene family in Haynaldia villosa demonstrated SnRK2.9-V enhances drought and salt stress tolerance of common wheat
Source: BMC Genomics. 2024 Feb 26;25:209. doi: 10.1186/s12864-024-10114-7 (PMC10895793; doi:10.1186/s12864-024-10114-7)
Supplement: Supplementary file 2 — Supplementary Material 2. [file 12864_2024_10114_MOESM2_ESM.doc]

**Supplementary Information**

Table S1. Characteristics of *SnRK2* gene family members in Triticeae Species.

| Gene name | Gene ID | Protein length(aa) | CDS length(bp) | Isoelectric Point | Molecular Weight(kDa) | Predicted subcellular location | Gene position |
| --- | --- | --- | --- | --- | --- | --- | --- |
| SnRK2.1-V | Dv02G537000.1 | 343 | 1029 | 5.47 | 38.89 | cytoplasm and nucleus | chr2V：599613766—599618016 |
| SnRK2.2-V | Dv02G177100.1 | 353 | 1059 | 5.62 | 40 | cytoplasm and nucleus | chr2V：81556836—81561619 |
| SnRK2.3-V | Dv01G260700.1 | 342 | 1026 | 5.52 | 38.67 | cytoplasm and nucleus | chr1V：319441915—319444790 |
| SnRK2.4-V | Dv03G420500.1 | 363 | 1089 | 5.54 | 41.64 | cytoplasm and nucleus | chr3V：596538422—596543038 |
| SnRK2.5-V | Dv02G631300.1 | 393 | 1179 | 6.14 | 44.07 | cytoplasm and nucleus | chr2V：631009396—631011256 |
| SnRK2.6-V | Dv01G317500.1 | 360 | 1080 | 5.88 | 41.21 | cytoplasm and nucleus | chr1V：375268507—375272560 |
| SnRK2.7-V | Dv02G337800.1 | 357 | 1071 | 5.54 | 40.93 | cytoplasm and nucleus | chr2V：455455518—455460437 |
| SnRK2.8-V | Dv05G438700.1 | 366 | 1098 | 4.87 | 41.59 | cytoplasm and nucleus | chr5V：464584888—464591198 |
| SnRK2.9-V | Dv05G085200.1 | 361 | 1083 | 5 | 40.39 | cytoplasm and nucleus | chr5V：55929343—55931966 |
| SnRK2.10-V | Dv04G074500.1 | 361 | 1083 | 4.8 | 40.66 | cytoplasm and nucleus | chr4V：375268507—375272560 |
| TaSnRK2.1-A | TraesCS2A02G493800.1 | 342 | 1026 | 5.59 | 38.79 | cytoplasm and nucleus | chr2A：726029783—726034011 |
| TaSnRK2.1-B | TraesCS2B02G521800.1 | 343 | 1029 | 5.47 | 39 | cytoplasm and nucleus | chr2B：716809651—716816243 |
| TaSnRK2.1-D | TraesCS2D02G493700.1 | 342 | 1026 | 5.66 | 38.85 | cytoplasm and nucleus | chr2D：591027189—591031752 |
| TaSnRK2.2-A | TraesCS2A02G163800.1 | 353 | 1059 | 5.53 | 40 | cytoplasm and nucleus | chr2A：116013921—116019018 |
| TaSnRK2.2-B | TraesCS2B02G189600.1 | 353 | 1059 | 5.62 | 40.01 | cytoplasm and nucleus | chr2B：165115341—165119793 |
| TaSnRK2.2-D | TraesCS2D02G170700.1 | 341 | 1023 | 5.45 | 38.66 | cytoplasm and nucleus | chr2D：114550221—114555142 |
| TaSnRK2.3-A | TraesCS1A02G215900.1 | 342 | 1026 | 5.43 | 38.61 | cytoplasm and nucleus | chr1A：381819326—381822599 |
| TaSnRK2.3-B | TraesCS1B02G229400.1 | 342 | 1026 | 5.52 | 38.66 | cytoplasm and nucleus | chr1B：411987863—411990884 |
| TaSnRK2.3-D | TraesCS1D02G218200.1 | 342 | 1026 | 5.73 | 38.55 | cytoplasm and nucleus | chr1D：304838300—304841343 |
| TaSnRK2.4-A | TraesCS3A02G381100.1 | 363 | 1089 | 5.77 | 42.1 | cytoplasm and nucleus | chr3A：631575444—631579962 |
| TaSnRK2.4-B | TraesCS3B02G413800.1 | 363 | 1089 | 5.86 | 42.18 | cytoplasm and nucleus | chr3B：650797001—650801750 |
| TaSnRK2.4-D | TraesCS3D02G374300.1 | 363 | 1089 | 5.86 | 42.18 | cytoplasm and nucleus | chr3D：487807055—487811579 |
| TaSnRK2.5-A | TraesCS2A02G566700.1 | 391 | 1173 | 6.15 | 43.97 | cytoplasm and nucleus | chr2A：765760972—765762877 |
| TaSnRK2.5-B | TraesCS2B02G630000.1 | 394 | 1182 | 6.15 | 44.21 | cytoplasm and nucleus | chr2B：801162070—801163970 |
| TaSnRK2.5-D | TraesCS2D02G577000.1 | 391 | 1173 | 6.15 | 43.93 | cytoplasm and nucleus | chr2D：640297481—640299386 |
| TaSnRK2.6-A | TraesCS1A02G270800.1 | 360 | 1080 | 5.48 | 41.22 | cytoplasm and nucleus | chr1A：464837012—464840398 |
| TaSnRK2.6-B | TraesCS1B02G281100.1 | 360 | 1080 | 5.56 | 41.27 | cytoplasm and nucleus | chr1B：489249385—489253102 |
| TaSnRK2.6-D | TraesCS1D02G271000.1 | 360 | 1080 | 5.48 | 42.39 | cytoplasm and nucleus | chr1D：365727612—365731332 |
| TaSnRK2.7-A | TraesCS2A02G303900.1 | 357 | 1071 | 5.55 | 40.91 | cytoplasm and nucleus | chr2A：522072400—522077216 |
| TaSnRK2.7-B | TraesCS2B02G320500.1 | 357 | 1071 | 5.55 | 40.91 | cytoplasm and nucleus | chr2B：457108855—457113685 |
| TaSnRK2.7-D | TraesCS2D02G302500.1 | 357 | 1071 | 5.55 | 40.94 | cytoplasm and nucleus | chr2D：386248395—386253037 |
| TaSnRK2.8-A | TraesCS5A02G401700.1 | 366 | 1098 | 4.87 | 41.59 | cytoplasm and nucleus | chr5A：594570844—594577495 |
| TaSnRK2.8-B | TraesCS5B02G406400.1 | 366 | 1098 | 4.87 | 41.59 | cytoplasm and nucleus | chr5B：582578186—582584645 |
| TaSnRK2.8-D | TraesCS5D02G411900.1 | 366 | 1098 | 4.87 | 41.59 | cytoplasm and nucleus | chr5D：475302359—475309094 |
| TaSnRK2.9-A | TraesCS5A02G069500.1 | 360 | 1080 | 4.94 | 40.33 | cytoplasm and nucleus | chr5A：78157536—78160032 |
| TaSnRK2.9-B | TraesCS5B02G075800.1 | 363 | 1089 | 4.89 | 40.66 | cytoplasm and nucleus | chr5B：90507294—90509733 |
| TaSnRK2.9-D | TraesCS5D02G081700.1 | 364 | 1092 | 4.89 | 40.59 | cytoplasm and nucleus | chr5D：82186877—82189457 |
| TaSnRK2.10-A | TraesCS4A02G235600.1 | 361 | 1083 | 4.8 | 40.65 | cytoplasm and nucleus | chr4A：544198215—544201187 |
| TaSnRK2.10-B | TraesCS4B02G079300.1 | 361 | 1083 | 4.8 | 40.65 | cytoplasm and nucleus | chr4B：76816569—76819507 |
| TaSnRK2.10-D | TraesCS4D02G078100.1 | 361 | 1083 | 4.8 | 40.65 | cytoplasm and nucleus | chr4D：52470433—52473315 |
| TdSnRK2.1-A | TRIDC2AG069670.3 | 342 | 1026 | 5.69 | 38.73 | cytoplasm and nucleus | chr2A：718786123—718788715 |
| TdSnRK2.1-B | TRIDC2BG075290.1 | 346 | 1038 | 5.31 | 39.36 | cytoplasm and nucleus | chr2B：712054796—712061626 |
| TdSnRK2.2-A | TRIDC2AG020700.3 | 353 | 1059 | 5.53 | 40 | cytoplasm and nucleus | chr2A：119364089—119368364 |
| TdSnRK2.2-B | TRIDC2BG024600.6 | 353 | 1059 | 5.62 | 40.01 | cytoplasm and nucleus | chr2B：169938286—169942471 |
| TdSnRK2.3-A | TRIDC1AG032610.4 | 342 | 1026 | 5.43 | 38.64 | cytoplasm and nucleus | chr1A：383453833—383456581 |
| TdSnRK2.3-B | TRIDC1BG037950.1 | 342 | 1026 | 5.52 | 38.66 | cytoplasm and nucleus | chr1B：418322258—418325258 |
| TdSnRK2.4-A | TRIDC3AG054690.4 | 363 | 1089 | 5.93 | 43.26 | cytoplasm and nucleus | chr3A：627856844—627860838 |
| TdSnRK2.4-B | TRIDC3BG061270.10 | 363 | 1089 | 5.96 | 41.43 | cytoplasm and nucleus | chr3B：661741472—661745983 |
| TdSnRK2.5-A | TRIDC2AG081240.6 | 364 | 1092 | 5.95 | 41.83 | cytoplasm and nucleus | chr2A：769646866—769648221 |
| TdSnRK2.5-B | TRIDC2BG089810.4 | 375 | 1125 | 6.15 | 42.68 | cytoplasm and nucleus | chr2B：796336735—796338112 |
| TdSnRK2.6-A | TRIDC1AG040320.1 | 360 | 1080 | 5.49 | 42.11 | cytoplasm and nucleus | chr1A：467302873—467306511 |
| TdSnRK2.6-B | TRIDC1BG045820.2 | 340 | 1020 | 5.64 | 38.87 | cytoplasm and nucleus | chr1B：495388308—495391788 |
| TdSnRK2.7-A | TRIDC2AG044100.2 | 357 | 1071 | 5.64 | 41.95 | cytoplasm and nucleus | chr2A：517817748—517822269 |
| TdSnRK2.7-B | TRIDC2BG046790.1 | 357 | 1071 | 5.55 | 40.91 | cytoplasm and nucleus | chr2B：455850358—455855275 |
| TdSnRK2.8-A | TRIDC5AG058380.1 | 366 | 1098 | 4.87 | 41.59 | cytoplasm and nucleus | chr5A：589736104—589739794 |
| TdSnRK2.8-B | TRIDC5BG062540.2 | 366 | 1098 | 4.87 | 41.59 | cytoplasm and nucleus | chr5B：588522769—588526730 |
| TdSnRK2.9-A | TRIDC5AG010550.2 | 329 | 987 | 4.79 | 37.04 | cytoplasm and nucleus | chr5A：77183704—77185807 |
| TdSnRK2.9-B | TRIDC5BG012340.1 | 367 | 1101 | 5.05 | 40.87 | cytoplasm and nucleus | chr5B：96803758—96805829 |
| TdSnRK2.10-A | TRIDC4AG037120.1 | 361 | 1083 | 4.87 | 41.76 | cytoplasm and nucleus | chr4A：536516891—536519240 |
| TdSnRK2.10-B | TRIDC4BG012280.2 | 324 | 972 | 4.85 | 36.71 | cytoplasm and nucleus | chr4B：74192523—74195024 |
| TuSnRK2.1 | TuG1812G0200005559.01.T01 | 342 | 1026 | 5.59 | 38.79 | cytoplasm and nucleus | chr2A：713465590—713469802 |
| TuSnRK2.2 | TuG1812G0200001716.01.T01 | 341 | 1023 | 5.45 | 38.65 | cytoplasm and nucleus | chr2A：111237234—111241953 |
| TuSnRK2.3 | TuG1812S0001294800.01.T01 | 342 | 1026 | 5.43 | 38.58 | cytoplasm and nucleus | Tu_contig_5227：1845—4943 |
| TuSnRK2.6 | TuG1812G0100003090.01.T02 | 360 | 1080 | 5.48 | 42.42 | cytoplasm and nucleus | chr1A：464339420—464343171 |
| TuSnRK2.7 | TuG1812G0200003486.01.T02 | 357 | 1071 | 8.7 | 40.87 | cytoplasm and nucleus | chr2A：506257655—506262336 |
| TuSnRK2.8 | TuG1812S0002912400.01.T01 | 217 | 651 | 7.11 | 33.15 | cytoplasm and nucleus | Tu_contig_6544：1—5798 |
| TuSnRK2.9 | TuG1812G0500000817.01.T01 | 360 | 1080 | 4.94 | 40.37 | cytoplasm and nucleus | chr5A：80962489—80964948 |
| TuSnRK2.10 | TuG1812G0400000763.01.T01 | 361 | 1083 | 4.8 | 40.65 | cytoplasm and nucleus | chr4A：60731366—60734049 |
| AetSnRK2.1 | AET2Gv210870004 | 342 | 1026 | 5.66 | 38.81 | cytoplasm and nucleus | chr2D：589000291—589004954 |
| AetSnRK2.2 | AET2Gv203396006 | 353 | 1059 | 5.53 | 40.04 | cytoplasm and nucleus | chr2D：115620726—115624835 |
| AetSnRK2.3 | AET1Gv205442002 | 342 | 1026 | 5.62 | 38.63 | cytoplasm and nucleus | chr1D：310386963—310390071 |
| AetSnRK2.4 | AET3Gv208493003 | 363 | 1089 | 5.86 | 42.17 | cytoplasm and nucleus | chr3D：495821040—495825531 |
| AetSnRK2.5 | AET2Gv212874004 | 380 | 1140 | 6.15 | 42.77 | cytoplasm and nucleus | chr2D：645838560—645840614 |
| AetSnRK2.6 | AET1Gv206525004 | 360 | 1080 | 5.48 | 41.2 | cytoplasm and nucleus | chr1D：371638241—371641949 |
| AetSnRK2.7 | AET2Gv206878005 | 357 | 1071 | 5.55 | 40.93 | cytoplasm and nucleus | chr2D：385009534—385014093 |
| AetSnRK2.8 | AET5Gv209282003 | 366 | 1098 | 4.87 | 41.59 | cytoplasm and nucleus | chr5D：484964704—484971188 |
| AetSnRK2.9 | AET5Gv201967001 | 364 | 1092 | 4.89 | 40.58 | cytoplasm and nucleus | chr5D：85622265—85624818 |
| AetSnRK2.10 | AET4Gv201653004 | 390 | 1170 | 4.75 | 44.11 | cytoplasm and nucleus | chr4D：55321222—55324184 |
| AesSnRK2.1 | AE.SPELTOIDES.r1.2BG0181750.1 | 343 | 1029 | 5.47 | 38.98 | cytoplasm and nucleus | chr2B：606704121—606708388 |
| AesSnRK2.2 | AE.SPELTOIDES.r1.2BG0113650.1 | 341 | 1023 | 5.45 | 38.63 | cytoplasm and nucleus | chr2B：135494413—135498568 |
| AesSnRK2.3 | AE.SPELTOIDES.r1.1BG0040060.1 | 342 | 1026 | 5.52 | 38.66 | cytoplasm and nucleus | chr1B：288974355—288976924 |
| AesSnRK2.4 | AE.SPELTOIDES.r1.3BG0275940.1 | 363 | 1089 | 5.86 | 42.12 | cytoplasm and nucleus | chr3B：503164763—503169005 |
| AesSnRK2.5 | AE.SPELTOIDES.r1.2BG0199220.1 | 394 | 1182 | 6.15 | 44.14 | cytoplasm and nucleus | chr2B：657280183—657281556 |
| AesSnRK2.6 | AE.SPELTOIDES.r1.1BG0047960.1 | 371 | 1113 | 5.56 | 42.37 | cytoplasm and nucleus | chr1B：339346822—339350187 |
| AesSnRK2.7 | AE.SPELTOIDES.r1.2BG0144900.1 | 359 | 1077 | 5.64 | 41.16 | cytoplasm and nucleus | chr2B：421046874—421051633 |
| AesSnRK2.8 | AE.SPELTOIDES.r1.5BG0472080.1 | 366 | 1098 | 4.87 | 41.59 | cytoplasm and nucleus | chr5B：516524345—516531063 |
| AesSnRK2.9 | AE.SPELTOIDES.r1.5BG0404010.1 | 363 | 1089 | 4.94 | 40.63 | cytoplasm and nucleus | chr5B：93634368—93636372 |
| AesSnRK2.10 | AE.SPELTOIDES.r1.4BG0326600.1 | 361 | 1083 | 4.8 | 40.64 | cytoplasm and nucleus | chr4B：56953093—56955345 |
| HvSnRK2.1 | HORVU2Hr1G110230.2 | 342 | 1026 | 5.75 | 38.82 | cytoplasm and nucleus | chr2H：719150904—719161174 |
| HvSnRK2.2 | HORVU2Hr1G029900.2 | 341 | 1023 | 5.45 | 38.59 | cytoplasm and nucleus | chr2H：108668003—108672779 |
| HvSnRK2.3 | HORVU1Hr1G055340.2 | 342 | 1026 | 5.85 | 38.53 | cytoplasm and nucleus | chr1H：405714931—405718538 |
| HvSnRK2.4 | HORVU3Hr1G082690.3 | 363 | 1089 | 5.96 | 41.93 | cytoplasm and nucleus | chr3H：600014204—600018365 |
| HvSnRK2.5 | HORVU2Hr1G125950.3 | 344 | 1032 | 5.85 | 39.19 | cytoplasm and nucleus | chr2H：762152464—762153836 |
| HvSnRK2.6 | HORVU1Hr1G074670.8 | 360 | 1080 | 6.12 | 47.29 | cytoplasm and nucleus | chr1H：508789881—508794175 |
| HvSnRK2.7 | HORVU2Hr1G075470.2 | 357 | 1071 | 5.54 | 40.9 | cytoplasm and nucleus | chr2H：543955717—543960490 |
| HvSnRK2.8 | HORVU5Hr1G097630.1 | 366 | 1098 | 4.86 | 41.53 | cytoplasm and nucleus | chr5H：605102179—605108556 |
| HvSnRK2.9 | HORVU5Hr1G018340.2 | 371 | 1113 | 4.99 | 41.33 | cytoplasm and nucleus | chr5H：74272143—74274322 |
| HvSnRK2.10 | HORVU4Hr1G013540.5 | 361 | 1083 | 4.8 | 40.63 | cytoplasm and nucleus | chr4H：47804453—47807197 |

*Table S2. Numbers of the ABRE, TGA-element, TATC-box, TGACG-motif, TCA-element, TC-rich repeats and LTR in SnRK2-V genes*.

| Gene Name | ABRE | TGA-element | TATC-box | TGACG-motif | TCA-element | TC-rich repeats | LTR |
| --- | --- | --- | --- | --- | --- | --- | --- |
| *SnRK2.1-V* | 2 | 0 | 0 | 2 | 1 | 0 | 1 |
| *SnRK2.2-V* | 3 | 0 | 0 | 6 | 2 | 0 | 0 |
| *SnRK2.3-V* | 2 | 0 | 0 | 1 | 0 | 0 | 0 |
| *SnRK2.4-V* | 0 | 0 | 0 | 1 | 1 | 0 | 0 |
| *SnRK2.5-V* | 3 | 0 | 0 | 4 | 0 | 0 | 1 |
| *SnRK2.6-V* | 2 | 0 | 0 | 1 | 0 | 0 | 0 |
| *SnRK2.7-V* | 3 | 3 | 0 | 3 | 0 | 0 | 0 |
| *SnRK2.8-V* | 0 | 0 | 0 | 1 | 1 | 0 | 1 |
| *SnRK2.9-V* | 5 | 0 | 0 | 0 | 0 | 1 | 2 |
| *SnRK2.10-V* | 2 | 0 | 0 | 1 | 0 | 0 | 0 |

Table S3. The name, sequence of the primer pairs.

| Primer name | Primers sequence ( 5'-3' ) | Application |
| --- | --- | --- |
| 2.1-1305-F： | CAGCCCAGATCAACTAGTATGGATCGGTACGAGGTG | Subcellular localization Primers for SnRK2.1-V |
| 2.1-1305-R： | CTTGCTCACCATGGATCCCAACGGGCACACGAAA |
| 2.2-1305-F： | CAGCCCAGATCAACTAGTATGGAGCGGTACGAGGT | Subcellular localization Primers for SnRK2.2-V |
| 2.2-1305-R： | CTTGCTCACCATGGATCCCAACGCGCACACGAA |
| 2.3-1305-F： | CAGCCCAGATCAACTAGTATGGAGGAGAGGTACGAGGCGTT | Subcellular localization Primers for SnRK2.3-V |
| 2.3-1305-R： | CTTGCTCACCATGGATCCGTAGGTCTCCCCCTCGGCTCC |
| 2.4-1305-F： | CAGCCCAGATCAACTAGTATGGAGAAGTACGAGGCGGTGC | Subcellular localization Primers for SnRK2.4-V |
| 2.4-1305-R： | CTTGCTCACCATGGATCCTGATATGCGTAGCGAGCTCATG |
| 2.5-1305-F： | CAGCCCAGATCAACTAGTATGGAGAAGTACGAGCCGGTAAG | Subcellular localization Primers for SnRK2.5-V |
| 2.5-1305-R： | CTTGCTCACCATGGATCCGATTTGGAGCTTGCTCATGCC |
| 2.6-1305-F： | CAGCCCAGATCAACTAGTATGGACAAGTACGAGG | Subcellular localization Primers for SnRK2.6-V |
| 2.6-1305-R： | CTTGCTCACCATGGATCCGATGTGTAACGCGCTCATATCC |
| 2.7-1305-F： | CAGCCCAGATCAACTAGTATGGAGAGGTACGAACTG | Subcellular localization Primers for SnRK2.7-V |
| 2.7-1305-R： | CTTGCTCACCATGGATCCGCTGATGTGGAACTCA |
| 2.8-1305-F： | CAGCCCAGATCAACTAGTATGGCAGGGGCGGCGCCG | Subcellular localization Primers for SnRK2.8-V |
| 2.8-1305-R： | CTTGCTCACCATGGATCCCATCGCATACACGATCT |
| 2.9-1305-F： | CAGCCCAGATCAACTAGTATGGAGAGGGGGCCGATGGCGGG | Subcellular localization Primers for SnRK2.9-V |
| 2.9-1305-R： | CTTGCTCACCATGGATCCCATGGCGTATACTATCTCCCCG |
| 2.10-1305-F： | CAGCCCAGATCAACTAGTATGGACCGGGCGGCGCTGACG | Subcellular localization Primers for SnRK2.10-V |
| 2.10-1305-R： | CTTGCTCACCATGGATCCCATAGCATACACTATCTC |
| Tublin-F | GATGCAGCCAACAACTTCGCC | qRT-PCR Primers for Tublin |
| Tublin-R | CAGTTCCACCTCCAACAGCGT |
| 2.1-Q-F： | TATGATGGAAAGGTCGC | qRT-PCR Primers for SnRK2.1-V |
| 2.1-Q-R： | AGACTCGAACGTAGTCCGGA |
| 2.2-Q-F： | CATTTGCTGTCCCGGATTTTCGT | qRT-PCR Primers for SnRK2.2-V |
| 2.2-Q-R： | CTGCGGGGGAGCGTTGACGCCGA |
| 2.3-Q-F： | TCCGACTGCAGACGCCTCCT | qRT-PCR Primers for SnRK2.3-V |
| 2.3-Q-R： | GCCCTGTCCCTCTCCGCGATCT |
| 2.4-Q-F： | GCGAGCTCGTCGCCGTCAAG | qRT-PCR Primers for SnRK2.4-V |
| 2.4-Q-R： | TCACAGATTCGCTCAAACAG |
| 2.5-Q-F： | GAGTACGTGCACGTCTCGCAGA | qRT-PCR Primers for SnRK2.5-V |
| 2.5-Q-R： | CGGCGGTTGTAGTAGGCCTGCT |
| 2.6-Q-F： | TGTGTACCGGGAGATCGTCAA | qRT-PCR Primers for SnRK2.6-V |
| 2.6-Q-R： | GTAAATTGACTCTGGATTTC |
| 2.7-Q-F： | CTCTCCCGCCGGGAATACGAC | qRT-PCR Primers for SnRK2.7-V |
| 2.7-Q-R： | AAGGAGTTGCTTGCAGTCTTG |
| 2.8-Q-F： | TAGTCCTGCTCCTCGCTTGAA | qRT-PCR Primers for SnRK2.8-V |
| 2.8-Q-R： | ACCGACTACCATGACATAA |
| 2.9-Q-F： | TCTGCTGGATGGAAGCACCG | qRT-PCR Primers for SnRK2.9-V |
| 2.9-Q-R： | CACGTAAAGGGTTACGCCACAT |
| 2.10-Q-F： | CGCTACGAGCTCGTCAA | qRT-PCR Primers for SnRK2.10-V |
| 2.10-Q-R： | CGTACTCCATGACGATGGCGA |
| TaSOD-F | CCTACTGGATGAGACGGAGAG | qRT-PCR Primers for TaSOD |
| TaSOD-R | GGACGAGGACAACGACGAA |
| TaCAT-F | CCATGAGATCAAGGCCATCT | qRT-PCR Primers for TaCAT |
| TaCAT-R | ATCTTACATGCTCGGCTTGG |
| TaAPX-F | TTGAGCCTATTAAAGCGAAGCA | qRT-PCR Primers for TaAPX |
| TaAPX-R | ACGGGGACAAACTGACGAA |
| TaPOD-F | GACAAGTCCCGTGTTGGTAGAG | qRT-PCR Primers for TaPOD |
| TaPOD-R | CGTGCAGCAACAGCCATTT |
| 110-2.9V-F1 | AGATTGCCGATGTGTGGTC | Identification Primers for SnRK2.9-V |
| 110-2.9V-R1 | AGGCTCCTCATACTGGTTGC |
| 110-ZT-2.9V-F1 | TCCCCGGGTACCGAGCTCATGGAGAGGGGGCCGA | Overexpression Vector Construction Primers for SnRK2.9-V |
| 110-ZT-2.9V-R1 | TCGGGGAAATTCGAGCTCCTACATGGCGTATACTAT |
| SnRK2.1-V-F | ATGGATCGGTACGAGGTG | Clone Primers for SnRK2.1-V |
| SnRK2.1-V-R | TTACAACGGGCACACGAAA |
| SnRK2.2-V-F | ATGGAGCGGTACGAGGT | Clone Primers for SnRK2.2-V |
| SnRK2.2-V-R | TCACAACGCGCACACGAA |
| SnRK2.3-V-F | ATGGAGGAGAGGTACGAGGCGTT | Clone Primers for SnRK2.3-V |
| SnRK2.3-V-R | TCAGTAGGTCTCCCCCTCGGCT |
| SnRK2.4-V-F | ATGGAGAAGTACGAGGCGGTGCGGGAC | Clone Primers for SnRK2.4-V |
| SnRK2.4-V-R | TCATGATATGCGTAGCGAGCTCATGCGGA |
| SnRK2.5-V-F | ATGGAGAAGTACGAGCCGGTAAGGGAGATCGGG | Clone Primers for SnRK2.5-V |
| SnRK2.5-V-R | TCAACTGATCGGGAGAGGGAGCATGGAG |
| SnRK2.6-V-F | ATGGACAAGTACGAGG | Clone Primers for SnRK2.6-V |
| SnRK2.6-V-R | TTAGATGTGTAACGCGCTCATATCC |
| SnRK2.7-V-F | ATGGAGAGGTACGAACTG | Clone Primers for SnRK2.7-V |
| SnRK2.7-V-R | CTAGCTGATGTGGAACTCA |
| SnRK2.8-V-F | ATGGCAGGGGCGGCGCCG | Clone Primers for SnRK2.8-V |
| SnRK2.8-V-R | TCACATCGCATACACGATCT |
| SnRK2.9-V-F | ATGGAGAGGGGGCCGATGGCGGGGCCGATGGGTGA | Clone Primers for SnRK2.9-V |
| SnRK2.9-V-R | CTACATGGCGTATACTATCTCCCCGCTGCTGTCCAT |
| SnRK2.10-V-F | ATGGACCGGGCGGCGCTGACG | Clone Primers for SnRK2.10-V |
| SnRK2.10-V-R | TCACATAGCATACACTATCTC |
